# Ligand-Dependent Conformations and Dynamics of the Serotonin 5-HT_2A_ Receptor Determine Its Activation and Membrane-Driven Oligomerization Properties

**DOI:** 10.1371/journal.pcbi.1002473

**Published:** 2012-04-19

**Authors:** Jufang Shan, George Khelashvili, Sayan Mondal, Ernest L. Mehler, Harel Weinstein

**Affiliations:** 1 Department of Physiology and Biophysics, Weill Medical College of Cornell University, New York, New York, United States of America; 2 The HRH Prince Alwaleed Bin Talal Bin Abdulaziz Alsaud Institute for Computational Biomedicine, Weill Medical College of Cornell University, New York, New York, United States of America; UNC Charlotte, United States of America

## Abstract

From computational simulations of a serotonin 2A receptor (5-HT_2A_R) model complexed with pharmacologically and structurally diverse ligands we identify different conformational states and dynamics adopted by the receptor bound to the full agonist 5-HT, the partial agonist LSD, and the inverse agonist Ketanserin. The results from the unbiased all-atom molecular dynamics (MD) simulations show that the three ligands affect differently the known GPCR activation elements including the toggle switch at W6.48, the changes in the ionic lock between E6.30 and R3.50 of the DRY motif in TM3, and the dynamics of the NPxxY motif in TM7. The computational results uncover a sequence of steps connecting these experimentally-identified elements of GPCR activation. The differences among the properties of the receptor molecule interacting with the ligands correlate with their distinct pharmacological properties. Combining these results with quantitative analysis of membrane deformation obtained with our new method (Mondal et al, Biophysical Journal 2011), we show that distinct conformational rearrangements produced by the three ligands also elicit different responses in the surrounding membrane. The differential reorganization of the receptor environment is reflected in (*i*)-the involvement of cholesterol in the activation of the 5-HT_2A_R, and (*ii*)-different extents and patterns of membrane deformations. These findings are discussed in the context of their likely functional consequences and a predicted mechanism of ligand-specific GPCR oligomerization.

## Author Summary

The 5-HT_2A_ receptor for the neurotransmitter serotonin (5-HT) belongs to family A (rhodopsin-like) G-protein coupled receptors (GPCRs), one of the most important classes of membrane proteins that are targeted by an extensive and diverse collection of external stimuli. Recently we learned that different ligands targeting the same GPCR can elicit different biological responses, but the mechanisms remain unknown. We address this fundamental question for the serotonin 5-HT_2A_ receptor, because it is known to respond to the binding of structurally diverse ligands by producing similar stimuli in the cell, and to the binding of quite similar ligands with dramatically different responses. Molecular dynamics simulations of molecular models of the serotonin 5-HT_2A_ receptor in complex with pharmacologically distinct ligands show the dynamic rearrangements of the receptor molecule to be different for these ligands, and the nature and extents of the rearrangements reflect the known pharmacological properties of the ligands as full, partial or inverse activators of the receptor. The different rearrangements of the receptor molecule are shown to produce different rearrangements of the surrounding membrane, a remodeling of the environment that can have differential ligand-determined effects on receptor function and association in the cell's membrane.

## Introduction

Serotonin 2A receptors (5-HT_2A_R) are a very well characterized family of G-protein coupled receptors (GPCRs) in the amine sub-class of rhodopsin-like class A GPCRs [1], [2]. The 5-HT_2A_Rs are targeted by chemically and pharmacologically distinct classes of ligands which include antidepressants, anxiolytics, antiemetics, antipsychotics and anti-migraine agents. Notably, some agonists exhibit hallucinogenic properties [2], [3] that have been attributed to specific manners of activation of these receptors [4], [5]. Even when they share key structural features, such as the indole moiety of the non-hallucinogen 5-HT and the hallucinogen LSD, the 5-HT_2A_R ligands have been shown to be able to bind differently to the receptor molecule, and to exhibit different pharmacological properties [2], [6], [7], [8]. Understanding the relation between the different modes of binding of structurally diverse compounds in the 5-HT_2A_R binding site, and the pharmacological responses they elicit, has therefore been of great interest in the quest for understanding the function of the 5-HT_2A_R and especially its role in hallucinogenesis [5]. Important clues came from in vivo studies demonstrating that behavioral responses to different 5-HT_2A_R ligands correlate with distinct transcriptome fingerprints for the ligands [4]. However, while it remains unclear how ligand binding induces distinct conformational states of the 5-HT_2A_R, and how this can result in different pharmacological outcomes [5], the significant variability in receptor conformations that can be induced by different ligands has recently been demonstrated for the cognate β_2_-adrenergic receptor [9].

**Figure 1:**
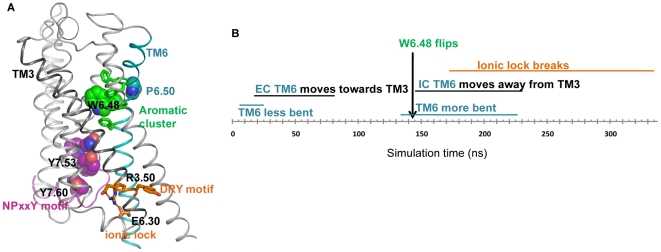
The position and dynamic sequence of Structural Motifs recognized as Functional Microdomains (SM/FMs) in the molecular model of the 5-HT_2A_R. (**A**) Known structural elements of GPCR activation (SM/FM) in the homology model of the **5-HT_2A_R**. (**B**) The time-ordered sequence of events identified from the MD simulations of the agonist-bound 5-HT_2A_R.

Structural evidence for differential effects of the GPCR ligands in relation to receptor function should be reflected in the variability of rearrangements in the key structural elements involved in the various activation states of the receptors, e.g., the structural motifs/functional microdomains (SM/FMs) [10] (see Figure 1A) that characterize GPCR activation [5], [11], [12], [13]. Specific SM/FMs have been reported from studies of a large variety of GPCRs [10], [14], [15], [16], [17], and their dynamic signatures include (*i*)-the flipping of the toggle switch W6.48 (Trp336, identified here by the Ballesteros-Weinstein generic numbering [18]) in the cluster of conserved aromatic residues in TM5 and TM6, (*ii*)-the opening/closing of the ionic lock between the DRY motif (D3.49–R3.50–Y3.51) and E6.30, involved in the movement of the intracellular (IC) end of TM6 away from TM3, and (*iii*)-the dynamics of the conserved NPxxY motif at the IC end of TM7 that connects as well to H8. These are elements of activation common to many GPCRs (see [5], [10], [11], [13], [14], [15], [16], [17], [19]), and their status in the X-ray structures of various GPCRs has been evaluated [12], [20], [21], [22], [23], [24], [25]. It is still unclear, however, how the binding of different ligands affects these elements of GPCR activation and how they connect to the mechanisms of the ligand-driven receptor oligomerization that has been shown to be critical for GPCR function [26], [27], [28], [29], [30], [31].

**Figure 2:**
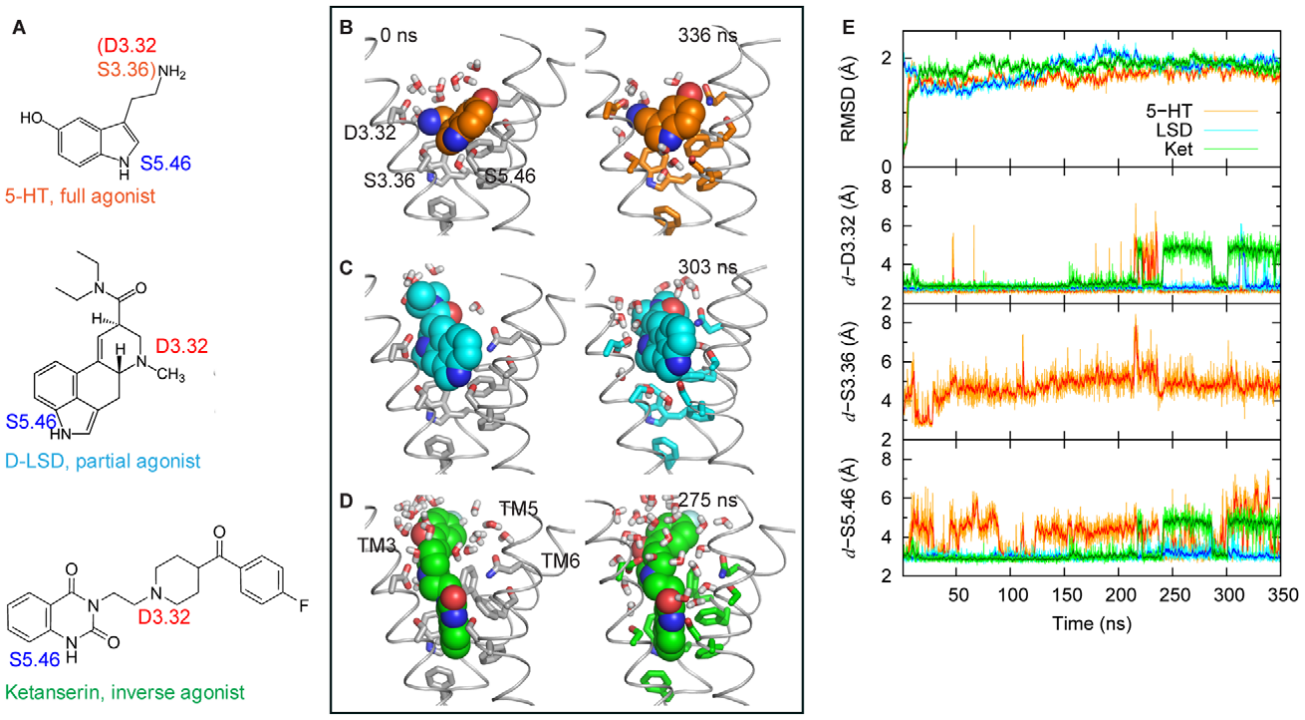
Structures of ligands with different efficacy and their interactions with 5-HT_2A_R during MD simulations. (**A**) Chemical structures of 5-HT, LSD and KET. Amines interacting with D3.32, S3.36 or S5.46 [6], [7], [116], [117], [118], [119] are labeled. (**B**,**C**,**D**) Docking poses in the initial structures (*left panels*) and during the simulations (*right panels*) for 5-HT (**B**), LSD (**C**) and KET (**D**), respectively. For clarity, only TM 3, 5 and 6 are shown in grey ribbons. Sidechains of residues D3.32, S3.36, S5.43, S5.46, F5.47, F6.44, W6.48, F6.51, F6.52 and N6.55 are depicted as sticks, and 5-HT (carbons colored in orange), LSD (cyan) and KET (green) are rendered in spheres. Note that, due to its large-size, and because its quinazoline ring penetrates deep into the binding pocket close to W6.48, KET is in direct contact with all the residues in the aromatic cluster, including F5.47. (**E**) Time-evolution of backbone TM RMSDs of 5-HT_2A_R (*upper panel*) and of the distances between the carboxyl/hydroxyl oxygens in D3.32, S3.36 and S5.46 on 5-HT_2A_R and their interacting amine nitrogens on ligands (see panel **A**) during the simulations (*lower panels*). Traces are shown in orange for 5-HT, in cyan for LSD, and in green for KET. Data were collected every 100 ps. Running averages were calculated every 10 data points and are shown in bold shades. N_α_ atom of 5-HT maintains a salt-bridge with D3.32 and forms an H-bond with S3.36 (Figure S2 in Text S1); N_1_ atom of 5-HT forms an H-bond with S5.46 either directly or through a water-bridge.

To shed new light on these central mechanistic questions from the perspective of ligand-dependent conformational states involved in the activation and oligomerization of GPCRs in their membrane environment, we performed large-scale molecular dynamics (MD) simulations of 5-HT_2A_R in complex with ligands exhibiting different pharmacological properties: the full agonist 5-HT, the partial agonist LSD, and the inverse agonist Ketanserin (KET) (Figure 2A). The simulation results show that the three ligands affect differently the dynamics of SM/FMs monitored in the simulations (Figure 1B), which achieve distinct conformations that are consistent with the pharmacological classification of these ligands. Moreover, the simulations show that the ligand-bound GPCRs produce differential responses in the lipid membrane surrounding the receptor, as reflected in the spatial pattern of the remodeling of membrane thickness. These trajectories reveal as well the modes and effects of direct receptor-cholesterol interaction. Recently we have described the development and implementation of a new method, CTMD (Combined conTinuum and Molecular Dynamics), for quantitative analysis of the membrane remodeling pattern based on MD trajectories [32]. With this method we account for both the membrane remodeling energy and the energy cost of any partial (incomplete) alleviation of the hydrophobic mismatch by this remodeling of the membrane. From the quantitative analysis with CTMD of the simulation results for the *monomeric* 5-HT_2A_R we identified ligand-specific local membrane perturbations that can produce different patterns of 5-HT_2A_R oligomerization driven by hydrophobic mismatch [32]. Our results lead to the prediction that the dimerization interfaces for 5-HT_2A_R oligomers will be different when the receptor binds ligands with different pharmacological properties (inverse agonist, partial agonist, or agonist), as suggested earlier [27]. Notably, the extent of membrane-driven oligomerization of a 5-HT_2A_R in the inverse agonist-bound state is predicted to be larger than in the agonist-bound state. These predictions are consistent with previous experimental findings on cognate GPCRs [27], [28], [31], supporting the link we identify here between ligand-dependent conformational changes in GPCRs and differences in local membrane perturbations.

## Results

### Agonist-determined dynamics are expressed as an ordered sequence of changes in the SM/FMs of ligand-bound 5-HT_2A_R

The main dynamic rearrangements observed in the simulations of the 5-HT_2A_R when it binds each of the ligands, are described below with reference to the SM/FMs (Figure 1A) identified in this family of GPCRs [5]. The sequential order of the description is determined by the order in which these changes appear in the simulation trajectories of the 5-HT_2A_R bound to the full agonist 5-HT (Figure 1B).

#### TM6 and the ionic lock

**Figure 3:**
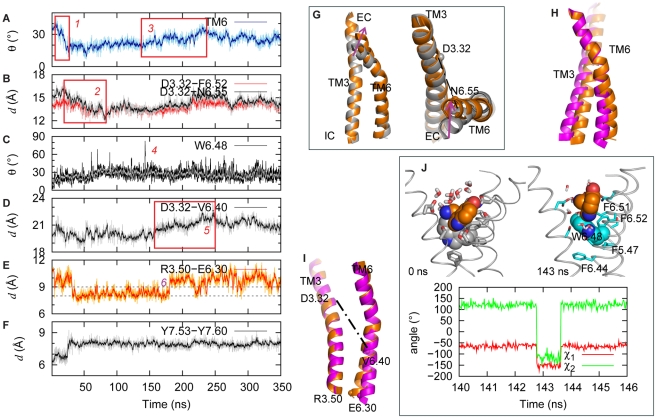
Activation steps of 5-HT_2A_R bound with 5-HT. (**A**) Evolution of the bend angle in TM6 around P6.50, highlighting the intervals during which the helix straightens (event *1*) and bends (event *3*) upon activation. (**B**) Evolution of the C_α_ distances between D3.32 and F6.52 (red), and between D3.32 and N6.55 (black), illustrating the interval during which the EC end of TM6 moves towards TM3 (event *2*). (**C**) Evolution of the tilt angle of the toggle switch W6.48 aromatic ring with respect to the membrane normal, showing the time point of W6.48 flipping (event *4*). W6.48 becomes parallel to the membrane for ∼1 ns at 143 ns (see panel **J**) with χ_1_ angle changing from *g-* to *trans*. (**D**) Evolution of the C_α_ distance between D3.32 and V6.40 illustrating the interval when the IC end of TM6 moves away from TM3 (event *5*). (**E**) Dynamics of the ionic lock presented as the evolution of the C_α_ distance between R3.50 and E6.30. Initially broken ionic lock forms during the first 50 ns, before opening again upon activation at ∼170 ns (event *6*). (**F**) Evolution of the C_α_ distance between Y7.53 and Y7.60. (**G**) Snapshots from the membrane plane and the EC end, highlighting positions of D3.32 and N6.55 and the distance between them, and showing the initial straightening and motion of TM6 towards TM3 (event *1*). Gray cartoon represents the starting structure, and the orange cartoon is the structure averaged over the 83–112 ns interval. (**H**) Cartoon representation of TM3 and TM6 highlighting the kink in the TM6 that occurs in the 135–225 ns time interval (event *3*). Orange and Magenta cartoons represent structures averaged over 83–112 ns and 290–350 ns, respectively. (**I**) Snapshots of TM3 and TM6 depicting positions of R3.50 and E6.30 residues and the distance between them and illustrating the movement of TM6 away from TM3 (event *5*). Color code is the same as in panel **G**. (**J**) Detailed dynamics in the toggle switch W6.48. Evolution of the χ_1_ and χ_2_ angles is shown during the 140–146 ns time-interval when the toggle switch flips. Also shown are the snapshots at 0 ns and 143 ns time-points of the 5-HT and W6.48 (in spheres and colored by atom type, 5-HT in orange, and W6.48 in grey and cyan at 0 ns and 143 ns, respectively.).

Figure 1 illustrates the known structural characteristics of GPCR activation and the time-ordered sequence of their occurrence in the simulation of the 5-HT-bound 5-HT_2A_R. The time-dependent changes in these SM/FMs are detailed in Figure 3. During the initial stages of the simulation, the changes in the orientation of the TM6 segments (before and after the Proline-kink) cause the bend to straighten out (Figure 3A) and the extracellular (EC) end of TM6 to move toward TM3 (Figure 3B and G). This is consistent with a conformational change observed in the crystal structure of β_2_AR [25] as well as in experiments [33] associated with β_2_AR activation. It is interesting to note that the change in bend angle around P6.50, from 33.2 in the inactive β_2_AR (2RH1_chain A) to 25.9 in the active β_2_AR (3SN6_chain R), is consistent with the first changes observed in the simulation of 5-HT-bound 5-HT_2A_R (Figure 3). In addition, the ionic lock (between the DRY motif on the IC end of TM3 and E6.30) changes as shown in Figure 3E: it equilibrates first into a closed form, but in later stages of the trajectory switches back to an open form compatible with the expected agonist-activated conformation; the IC end of TM6 moves away from TM3 (Figure 3D, I). This is remarkable because of the opening of the ionic lock between the DRY motif and E6.30 is a landmark of GPCR activation [10], [34], [35], [36], [37], and the broken ionic lock is evident in an active β_2_AR structure stabilized by nanobody [23] as well as an agonist-bound β_2_AR in complex with the nucleotide free Gs heterotrimer [25].

#### The aromatic cluster

From the trajectory, the opening of the ionic lock (Figure 3E) and the movement of the IC end of TM6 (Figure 3D) appear to relate to the rotamer flip of W6.48 from its orientation near-perpendicular to the membrane plane, to a near-parallel one (Figure 3C, J). Such a conformational switch in W6.48 upon GPCR activation has been reported from a variety of experimental studies [36], [38], and is observed near the 140 ns time point in the trajectory when the χ_1_ angle of W6.48 changes from *g-* to *trans* (Figures 1B,3C,3J). When the ring of W6.48 remains parallel to the bilayer for ∼1 ns it forms a double pi-pi interaction with both F6.44 and F6.51 (see Figure S1 in Text S1). This may facilitate the change in TM6 kink around P6.50 as suggested earlier [39] which would thus support the opening of the ionic lock by increasing the distance between the IC ends of TM6 and TM3 (Figure 3D).

#### The NPxxY motif and helix 8

During the first 50 ns of the agonist-bound 5-HT_2A_R simulation, the conserved NPxxY motif at the IC end of TM7 changes its conformation and spatial relation to H8 (Figure 3F). The dynamics in this SM/FM have been related to GPCR activation [19], [22]. In particular, the interdependence of residues at positions 7.53 and 7.60 in the NPxxY sequence has been suggested to modulate the transition to the active state in the serotonin 2C receptor (5-HT_2C_R) [24], and structural data show that the pi-pi interaction between 7.53 and 7.60 (Figure 1A) seen in inactive structures, is disrupted in active structures of β_2_AR stabilized by nanobody [23], or complexed with the Gs heterotrimer [25]. In addition, the C_α_ distances between 7.53 and 7.60 in these active structures are larger (9.6 ???) than those in inactive ones (6.3 ???). The opening of the TM7-H8 angle is consistent with the transition to activated states of other GPCRs [22], and here we find that, in the 5-HT-bound 5-HT_2A_R, H8 moves away from TM7 and the distance between the C_α_ atoms of Y7.53 and Y7.60 increases from ∼6.2 ??? during the first 50 ns, to a value of 8 Å that remains stable for the remainder of the trajectory (Figure 3F).

*Taken together, this sequence of steps observed in our simulations of 5-HT_2A_R in complex with 5-HT (* *Figure 1B* *) not only captures structural effects of agonist binding on the status of the SM/FMs, but also provides a mechanistically understandable hypothesis for this ordered sequence of apparently interrelated conformational changes that bring the 5-HT-bound 5-HT_2A_R in line with known features of the activated state.*

### Significant differences are evident in the dynamics of the same SM/FMs in 5-HT_2A_R bound to either the partial agonist LSD or the inverse agonist KET

**Figure 4:**
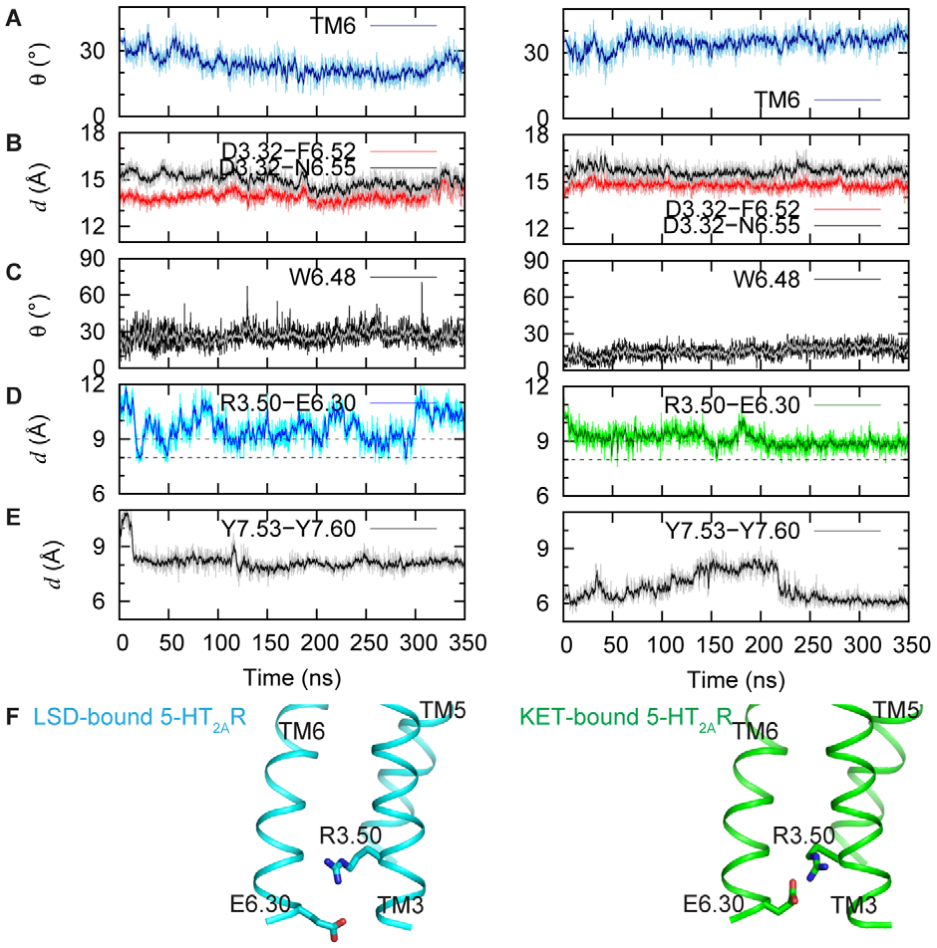
Dynamics of activation elements in LSD- and KET-bound 5-HT_2A_R. (**A–E**) Left and right panels show the evolution of active state components in the 5-HT_2A_R complexed with LSD and KET, respectively (for details see Figure 3). (**F**) Cartoon representation of TM3 and TM6 in the structures averaged over the last 100 ns of the LSD (cyan) and KET (green) trajectories, showing positions of R3.50 and E6.30 residues (in sticks).

Comparison of results in Figure 4 with Figure 3 brings to light the differences among the dynamic mechanisms connected with the binding of the three different ligands to the 5-HT_2A_R, as detailed below.

#### KET bound to the 5-HT_2A_R

In the KET simulation, the initially open ionic lock closes around 200 ns, and remains closed for the remainder of the trajectory, as the C_α_ distance between R3.50 and E6.30 residues stabilizes below 9 Å (Figure 4D, *right panel*), i.e., at a value consistent with inactive conformations of cognate GPCRs [40]. Similarly consistent with a preference for this SM/FM in an inactive form of the receptor, is the observation in KET-bound 5-HT_2A_R that neither the bend angle in TM6 (Figure 4A, *right panel*), nor the rotamer status of W6.48 (Figure 4C, *right panel*), change as they were seen to do in the trajectory of the 5-HT-bound receptor (Figure 3A, 3C). Thus, TM6 is more kinked with KET in the binding site than with 5-HT bound in the 5-HT_2A_R, but no movement of the EC end of TM6 is observed relative to TM3 (Figure 4B, *right panel*). This is in sharp contrast to the behavior of 5-HT-bound receptor (Figure 3B), where significant changes in these activation elements were observed. Further, the dynamics of the NPxxY motif is also different in the KET-bound receptor, with the TM7-H8 pair maintaining a tighter conformation, and the Y7.53-Y7.60 C_α_ distance stabilized at ∼6 Å for the later part of the trajectory (Figure 4E), i.e., 2 Å shorter than that in 5-HT_2A_R complexed with 5-HT (Figure 3F). Note that such close proximity of the Y7.53 and Y7.60 residues has been suggested as a characteristic of an inactive state in GPCRs [41].

**Figure 5:**
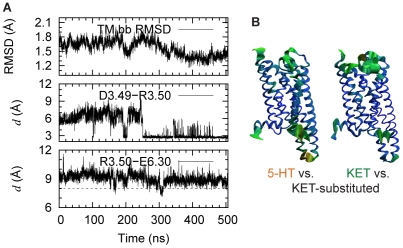
Characteristic dynamics of 5-HT_2A_R induced by 5-HT are reversed by KET. (**A**) Evolution of the TM backbone RMSD of KET-substituted receptor compared to KET-bound receptor, averaged along 250–350 ns (top), the minimum distance between the carboxylate oxygens of D3.49 and the guanidine nitrogens of R3.50 (middle), as well as the C_α_ distance between R3.50 and E6.30 (bottom). (**B**) Extreme projections along the first eigenvector from Comb-ED analysis of the combined 5-HT-bound and KET-substituted receptors (left panel), as well as KET-bound and KET-substituted (right panel) trajectories. The receptor is shown in tubes, and colors depict magnitudes of conformational changes from small to large (from blue to green, and to red).

To validate the inferences from the KET simulation, and verify the distinctions between the agonist-bound and inverse-agonist bound forms of the 5-HT_2A_R, we tested whether the binding of the inverse agonist KET would reverse the effect of the full agonist 5-HT on the conformational state of the serotonin receptor. To this end, we used the 5-HT-bound 5-HT_2A_R structure (from the average over the 300–350 ns trajectory interval of the simulation) as a starting structure for a new 500 ns simulation in which KET was substituted for the 5-HT (termed, KET-substituted, see Methods). As illustrated in Figure 5A, at ∼240 ns into this new trajectory, the ionic lock that had opened in the agonist-bound simulation started to close when KET replaced it (the E6.30–R3.50 C_α_ distance decreased below 9 Å), and D3.49 and R3.50 formed a salt-bridged H-bond. Furthermore, from the same time point onwards, the structure of the TM bundle gradually became more similar to that stabilized by KET (the backbone TM RMSD relative to the KET-stabilized structure decreased by ∼0.5 Å; Figure 5A).

*These results show that the SM/FMs in the 5-HT_2A_R bound to KET adopt characteristics observed in inactive GPCR states, which differ significantly from the ones observed with 5-HT in the binding site. This observation is in line with the opposite pharmacological properties of these two ligands.*

#### LSD bound to the 5-HT_2A_R

As shown in Figure 4 (*left panel*), the dynamic behavior of the LSD-bound receptor is in line with the pharmacological efficacy of LSD as a partial agonist, i.e., intermediate between those observed for the 5-HT- and KET-bound 5-HT_2A_R constructs. Thus, in the LSD-bound receptor the ionic lock transitions between open and closed conformations (Figure 4D, *left panel*), as the R3.50–E6.30 distance fluctuates in the range of values proposed [40] for open (>9 Å) and closed (<9 Å) ionic lock states. In the NPxxY motif region, the Y7.53–Y7.60 distance in the LSD-bound receptor remains in the range associated with an open conformation throughout the trajectory, similar to that in the 5-HT bound receptor (*cf.* Figures 3F and 4E). The bend angle around P6.50 in the LSD-bound receptor decreases from 35° to 15° (Figure 4A, *left panel*) with the EC end of TM6 bending towards the center of the protein bundle. However, the extracellular segment of TM6 does not come as close to TM3 as it does in the 5-HT simulation (cf. D3.32-F6.52 and D3.32-N6.55 in Figure 3B and Figure 4B, *left panel*).

Consistent with the incomplete opening of the ionic lock, the time-trace of the tilt angle of the W6.48 aromatic ring in LSD trajectory (Figure 4C, *left panel*) indicates as well that the dynamics of the toggle switch is intermediate: the complete flipping of W6.48, observed in the 5-HT simulation (Figures 3C, 3J), is replaced here by significant fluctuations in W6.48 orientation compared to the KET-bound 5-HT_2A_R (Figure 4C, *right panel*). *Thus, in monitoring the steps in the dynamic sequence we find for the partial agonist LSD a series of intermediate dynamic modes that turn on the SM/FM forms associated with an active conformation, but not necessarily all of them together (e.g., the TM7-H8 angle is wider, and the ionic lock is broken, but with a fluctuating, not flipped W6.48). Like the other two ligand types, the pattern generated by LSD is entirely consistent with its known pharmacological property.*

### The 5-HT_2A_R in complex with either 5-HT, LSD, or KET visits distinct conformational spaces

**Figure 6:**
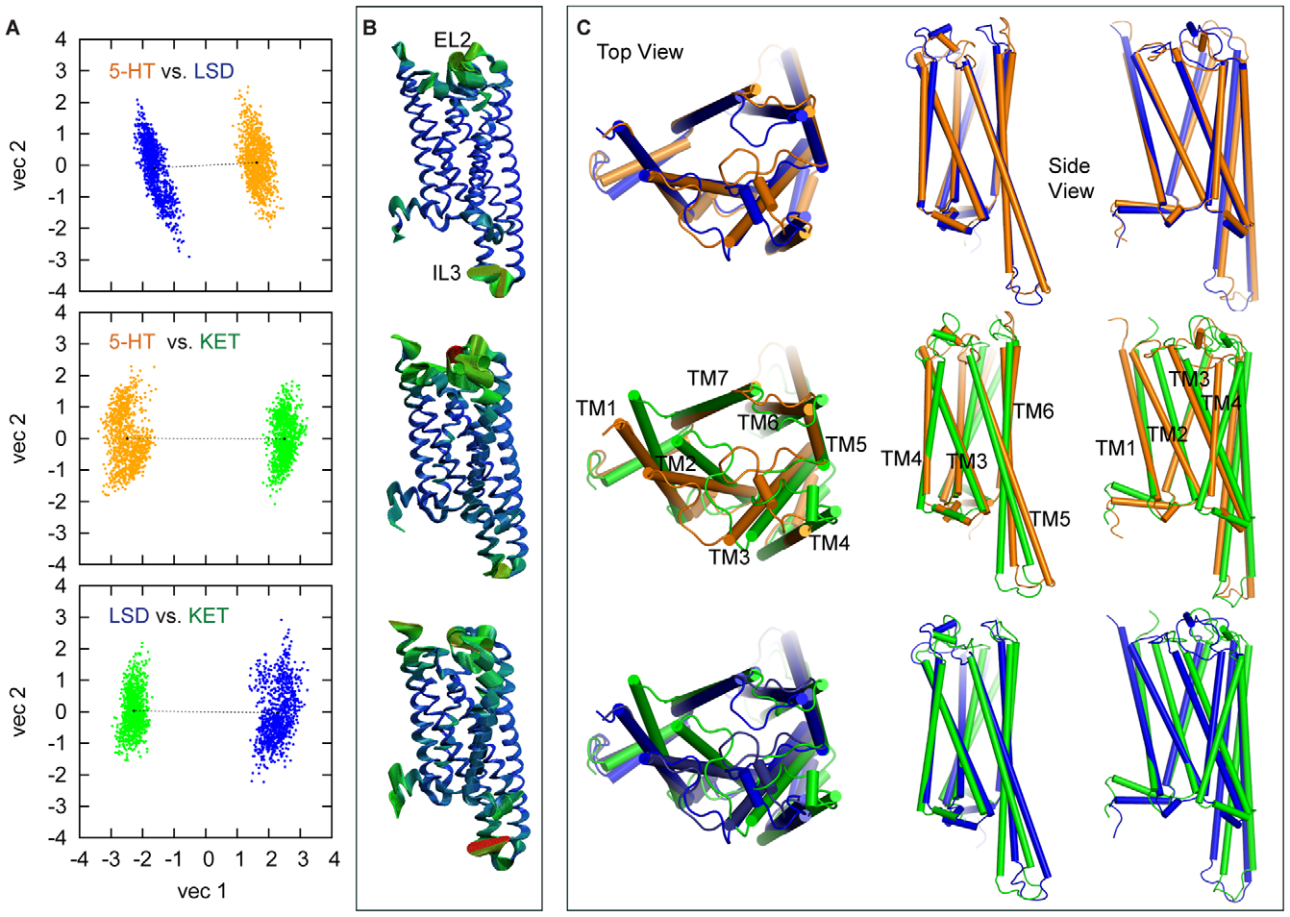
Comb-ED analysis of the conformational spaces visited by 5-HT_2A_R bound to 5-HT, LSD and KET. (**A**) Projections along the first and second eigenvectors obtained from the Comb-ED analysis on the concatenated 5-HT-LSD (upper panel), 5-HT-KET (middle panel), and LSD-KET (lower panel) trajectories. The centers of the conformational space sampled by ligands are in black dots and are connected by black dotted lines. (**B**) Extreme projections along the first eigenvector of the combined 5-HT-LSD (top panel), 5-HT-KET (middle panel) and LSD-KET (bottom panel) trajectories. The receptor is rendered and colored as in Figure 5B. (**C**) Comparison of the 5-HT_2A_R structures in complex with 5-HT, LSD or KET averaged over the final 100 ns aligned with seven most conserved residues in each TM [18]. The receptor structures in complex with different ligands are shown in cartoon and are colored as in panel **A**.

The nature of similarities and differences observed in the dynamics of the 5-HT_2A_R when it binds each of the three ligands was further evaluated with Combined Essential Dynamics (Comb-ED, see Methods) [42] performed on concatenated trajectories for 5-HT&LSD, 5-HT&KET, and LSD&KET, each combining the last 100 ns of the individual trajectories for the pair. The comparison of such combined trajectories by their projection along their first and second eigenvectors is shown in Figure 6A, which illustrates the differences in the conformational spaces sampled by the 5-HT_2A_R bound to different ligands. Clearly, along the first eigenvector, the conformational spaces sampled by the 5-HT-bound and LSD-bound receptor are seen to be more similar to each other than either one is to the space sampled by KET-bound 5-HT_2A_R (note that the first and second eigenvectors are different in each plot because the concatenated trajectories differ, so that the sampled spaces shown in the plots for any one ligand-bound receptor appear at different positions).

The comparison in Figure 6B–C shows the differences in a structural context by indicating where the largest differences occur, as monitored by the magnitudes of the projections on the first eigenvectors (color coded from red, green to blue representing magnitudes from large, median to small, respectively). Also evident in this figure is the greater similarity between the dynamics of the 5-HT and LSD-bound receptors (Figure 6B–C, *top panel*). Comb-ED analysis identifies only insignificant differences between the agonist- vs. partial agonist-bound states of the receptor, with some variations in the positioning of the juxta-membrane H8 and in TM4 (Figure 6B–C, *top panel*). However, the structure of 5-HT_2A_R in complex with either 5-HT or LSD is clearly distinct from that with KET bound, as seen in Figure 6B–C where the Comb-ED detects differences in TM5–6 (linked by IL3) and TM4 in the 5-HT vs. KET comparison (*middle panel*), and LSD vs. KET (*bottom panel*).

Differences between 5-HT_2A_R complexes with the inverse agonist, and those with the agonists 5-HT or LSD, are apparent as well for TM1, TM3 and H8 (Figure 6B–C, *middle and bottom panels*). Thus, in the KET-bound receptor, Comb-ED identifies the movement of TM5 and TM6 toward TM3 at the IC end, consistent with the observed closing of the ionic lock in the inverse agonist state (Figure 4D,F, *right panel*). Furthermore, differences are evident at the EC end of TM6 between KET- and 5-HT-induced conformations, in agreement with the different level of kink in TM6 around the P6.50 in the two systems (compare Figures 3A and 4A). In addition, in line with the observed differences in the dynamics of NPxxY motif (Figures 3F and 4E), the Comb-ED analysis in the KET-bound receptor detects the motion of H8 toward TM7 to close the angle between them, consistent with earlier studies of cognate GPCRs [22], [37], [43].

Based on the Comb-ED results suggesting structural differences as well in TM1 and TM4 between the states of 5-HT_2A_R stabilized by the three ligands (Figure 6B–C), we found different levels of tilt in TM1 and TM4 in the three states of the receptor. Thus, in 5-HT, LSD, and KET trajectories TM4 forms angles of 12°, 16° and 22°, respectively, with the membrane normal *z* axis; TM1 tilts so that in KET-bound compared to 5-HT-bound receptors its EC end is 3 Å closer to TM7 and its IC side is 1.5 Å farther from TM7. The differences in conformational changes of TM1 are consistent with the available X-ray structures of the activated GPCR, where a repositioning of the IC end of TM7 towards TM1 is reported in active β_2_AR [23] and opsin structures [20], [21]. As discussed below, these tilt differences in TM1 and TM4 are reflected in the response of the membrane to the interaction with the protein, and thereby can affect the ligand-regulated oligomerization of the 5-HT_2A_R.

The nature of the changes occurring in the transition from the “activated” 5-HT-bound state of the receptor, to the KET-bound “inactivated” state, is evidenced by the application of Comb-ED analysis to combined trajectories involving the KET-substituted simulation (started from an equilibrated 5-HT-bound receptor) (Figure 5B). Separately, two Comb-ED analysis were performed: One comparing the last 100 ns from the KET-substituted and the original KET-bound simulations, and the other comparing the KET-substituted and the 5-HT-bound simulation. The projections along the first eigenvector of these combined trajectories (Figure 5B) reveal the internal consistency of the results and show that, upon KET substitution, the 5-HT_2A_R structure deviated from the 5-HT-stabilized conformation and became similar to that stabilized by KET in our earlier simulation, with TM4 and TM6 helices changing the most. Consistent with the results in Figure 6, in the KET-substituted simulation the IC end of TM6 moved towards TM3, and TM4 became tilted.

In addition to Comb-ED analysis of pair-wise concatenated trajectories, we applied Comb-ED as well to all four trajectories (5-HT, LSD, KET, KET-substituted) concatenated together. The results (Figure S3 in Text S1) clearly show that KET-substitution transitions the receptor from the conformational states visited by 5-HT to those most visited when KET is bound in the receptor.

### Ligand-dependent conformational changes in the receptor elicit corresponding structural re-arrangements in the surrounding lipid membrane

From the results of the comparative simulations we have identified two mechanisms of membrane re-organization in response to the conformational changes associated with the dynamics of the ligand-bound receptor: (*i*)-the direct interactions of the receptor with the Cholesterol (Chol) constituent of the membrane, and (*ii*)-the deformation of the membrane around the GPCR, which modulates the local thickness of the bilayer and the hydrophobic mismatch that can drive oligomerization of the 5-HT_2A_R [32].

#### Cholesterol interacts with the structural elements of GPCR activation

Cholesterol has been implicated in GPCR function and activation [44] and shown to bind to preferred sites of rhodopsin in extended simulations of this GPCR in lipid membranes [43]. Here we found that the regions ranked highest in Chol population during the simulation of the 5-HT_2A_R were the IC end of a TM bundle including TMs 1, 2 and 4; the EC ends of TMs 2 and 3; and the EC and IC ends of TMs 6 and 7 (see Table S1 in Text S1). Notably, these sites were also found to be the areas of high Chol-density in earlier studies on rhodopsin [43], [45], [46], as well as in a 250 ns simulation of rhodopsin in a membrane with the same lipid composition as used here (SDPC/POPC/Chol - see [32]). We hypothesized, therefore, that Chol binding at these preferred locations may have some functional importance observable through effects on the monitored SM/FMs. Given the prominent structural changes in TM6 observed in our simulations of the 5-HT-bound 5-HT_2A_R, this hypothesis was investigated for Chol at the IC and EC ends of TMs 6 and 7.

**Figure 7:**
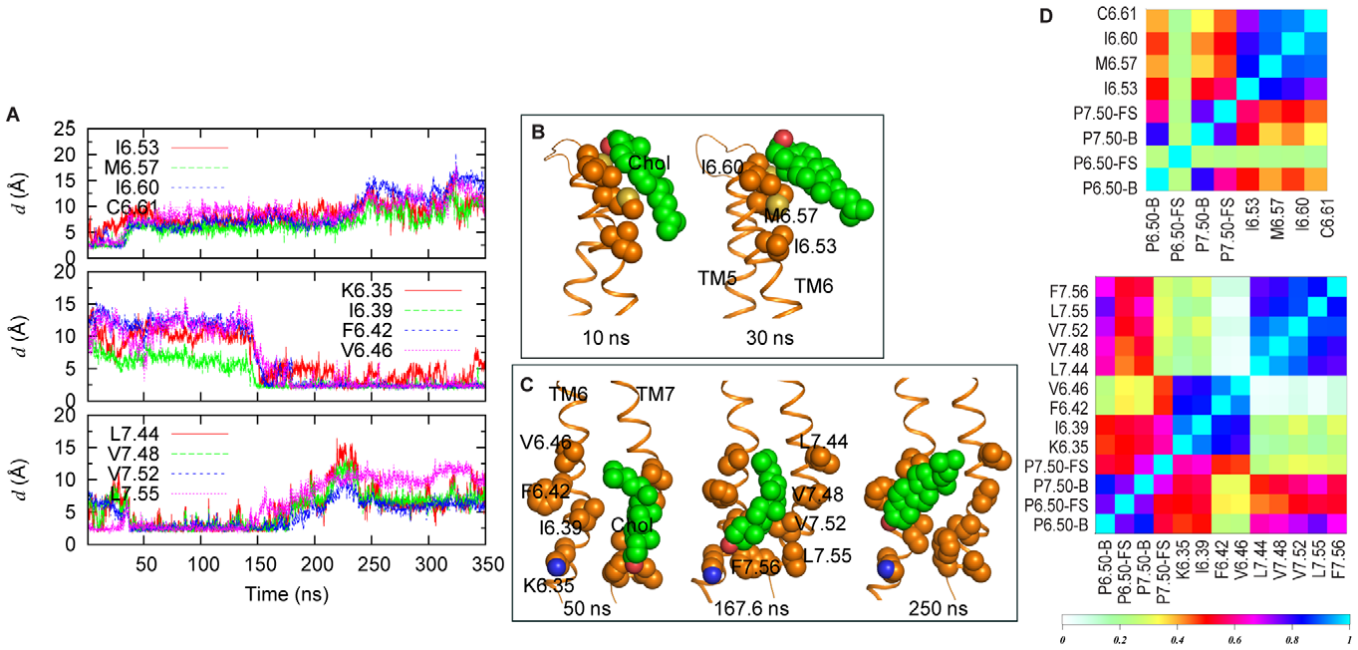
Cholesterol dynamics correlates with the structural transitions in agonist-bound 5-HT_2A_R. (**A**) Evolution of the minimum distances between the Chol at the EC end of TM6 and selected TM6 residues in the 5-HT simulation (top panel). Time traces of the minimum distances between the Chol at the IC ends of TM6–7 and selected residues on TM6 and 7 (middle and bottom panels). The Chol initially in contact with the L7.44, V7.48, V7.52, and L7.55 residues on TM7 moves towards TM6 and engages in interactions with the residues K6.35, I6.39, F6.42, and V6.46 on TM6. (**B**) Snapshots at 10 and 30 ns showing the Chol from the top panel of (**A**) interacting with EC TM6. (**C**) Snapshots at 50, 167.6 and 250 ns showing the Chol from the bottom panels of (**A**) interacting with either IC TM6 or IC TM7. (**D**) Matrix of Pearson's score tests performed on the dynamics quantities presented in the top panel of (A) and on the bend (“B”) and face-shift (“FS”) angles around P6.50 and P7.50 (top panel). Matrix of Pearson's score tests performed on the dynamic quantities presented in middle and bottom panels of (A) and on the bend (“B”), and face-shift (“FS”) angles of P6.50 and P7.50 (bottom panel).

Figure 7 summarizes Chol dynamics around the EC and IC ends of TMs 6 and 7, and its relation to the activation elements in 5-HT-bound receptor. The Chol at the EC end of TM6 is seen to be in direct contact with residues M6.57, I6.60, and C6.61 at the initial stages of the simulation (Figure 7A, *upper panel*), and to move away from these residues within the first 40 ns (Figure 7B). Interestingly, during the same time interval, we observe changes in one of the identified SM/FMs, as TM6 straightens out at the EC end, and starts moving towards TM3 (cf. Figures 2–3). Near the 140 ns time-point, another Chol, initially in contact with the IC end of TM7, moves towards TM6 and establishes interactions with residues K6.35, I6.39, F6.42 and V6.46 (Figure 7A, *middle and lower panels*, and Figure 7C). Remarkably, this shift of Chol away from TM7 and toward TM6 coincides with the time when the toggle switch W6.48 flips (Figures 2–3), and TM6 starts to bend away from TM3 at the IC end (Figures 2–3). *Thus, the time dependence of Chol dynamics at the IC and EC ends of TM6 suggests its participation in the development of the activated conformation in the agonist-bound 5-HT_2A_R.*

To quantify the apparent correlation between the Chol dynamics and the structural changes in the 5-HT/5-HT_2A_R simulation we calculated the Pearson correlation coefficients between the dynamic quantities presented in Figures 3 and 7, and constructed the matrix of the corresponding Pearson *R^2^* scores following a protocol described earlier [43]. The strong correlation between Chol-TM6 distances and agonist-induced changes in 5-HT_2A_R is evident from the high values of the correlation coefficients (Figure 7D) calculated for the MD trajectory.

Notably, the pattern of Chol-GPCR interactions around TM6 in the 5-HT/5-HT_2A_R trajectory is different from those observed in either the LSD-bound or the KET-bound receptor. For example, analysis of the KET simulation shows a single Chol around the IC ends of TM5 and TM6 (Table S1 and Figure S4 in Text S1). This Chol molecule is engaged in interactions with F6.42 and V6.46, the same two residues of TM6 that we found to interact as well with Chol in the 5-HT trajectory (see Figure 7). But in contrast to the 5-HT simulation, the cholesterol positioned near the IC end of TM6 in the KET trajectory is also in contact with residues from TM5: F5.59 and I5.62. The specificity of these interactions is suggested by the observation that in the KET-substitution simulation (initiated from the 5-HT-induced conformation of the receptor), Chol interactions rearrange to the pattern observed in the regular KET simulation. Thus, in the KET-substitution simulation Chol still interacts initially with F6.42, V6.46, and I6.39, but when the ionic lock starts to form, F6.42 flips (without loosing contact with the cholesterol) towards TM5 and establishes stable interactions with F5.59 that brings TM5 and TM6 helices together from the IC side. Similarly, in the KET simulation, cholesterol near TM5 and TM6 appears as well to bring these two helices together by forming a bridge between F6.42 and F5.59.

*When considered together with the ionic lock data (* *Figure 4* *), these results suggest an active involvement of cholesterol near the IC end of TM6 in establishing differential ligand-induced conformational dynamics of the receptor.*

### Membrane shape exhibits distinct patterns in response to the 5-HT_2A_R conformations attained by pharmacologically different ligands

**Figure 8:**
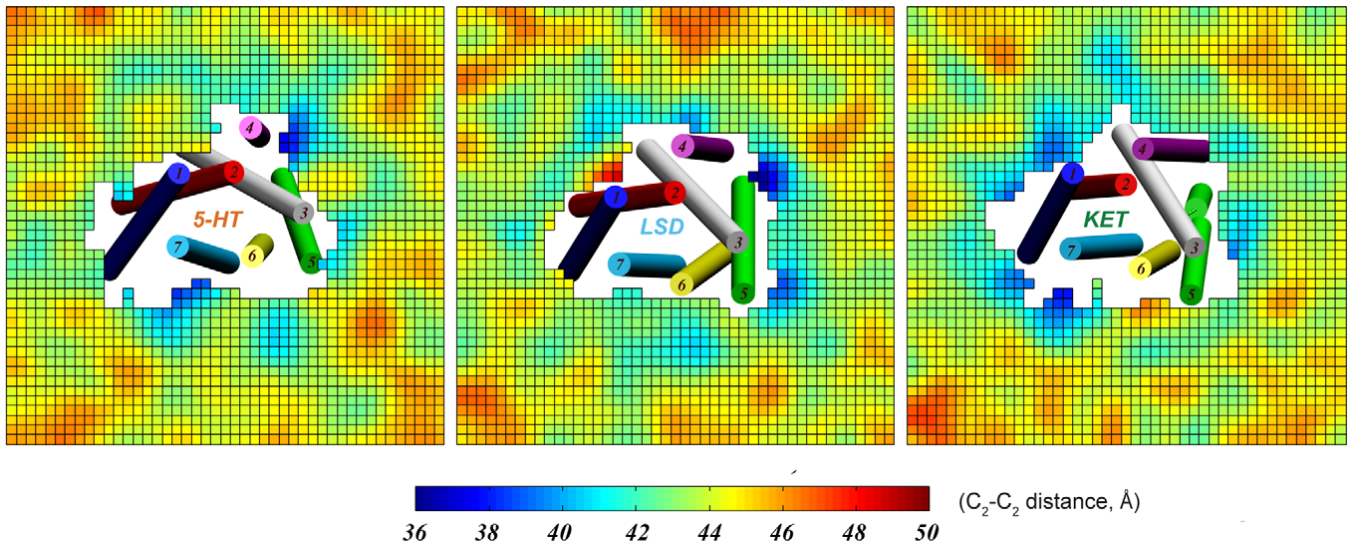
Hydrophobic thickness profiles of simulated membranes around 5-HT_2A_R in complex with 5-HT, LSD, or KET. The structures of the various ligand-bound receptor structures averaged over the last 100 ns of the simulations are shown in cartoon, with only the helices depicted (in different colors) with corresponding TM numbers. The colored fields represent distances (in Å) between lipid backbone C_2_ atoms from the opposing leaflets. For this analysis, for each simulated system the membrane plane was divided into square 2 Å×2 Å bins, and the average C_2_-C_2_ distances in each bin were collected by scanning the last 100 ns of trajectory.

The distinct conformational changes in the receptor produced by the binding of the different ligands (see above) produce different patterns of bilayer deformations around the receptor protein in complex with the different ligands (Figure 8). This difference is a result of the tendency of the lipids to minimize the hydrophobic mismatch at various TMs, i.e., the difference in the hydrophobic lengths presented to the membrane by the corresponding TMs in the different receptor complexes (see detailed discussion in [32]). Therefore, hydrophobic thickness profiles of membranes around 5-HT_2A_R in the simulated complexes with 5-HT, LSD, and KET, shown in Figure 8, reveal remarkable differences in the membrane organization around individual TMs in the three systems. For example, the membrane appears thinner around TM4 and TM6 in 5-HT (*left panel*) than in the KET simulation (*right panel*), whereas at TM1 the bilayer is thicker in the LSD (*middle panel*) than in the complexes with 5-HT or the KET.

We have developed a quantitative method (CTMD), for the analysis of such membrane deformations and the significant residual exposure to unfavorable hydrophobic-hydrophilic interactions at specific TMs that results from an incomplete alleviation of the hydrophobic mismatch [32]. When applied to the 5-HT_2A_R complexes discussed here, residual exposure [32] was found at TM4 for all three complexes, although the values were different possibly because the TM4 tilt is different in the KET, LSD and 5-HT trajectories (see above). Because the extent of the hydrophobic mismatch around the TM helices is considered to be a driving force for oligomerization [32], , we had compared the residual exposure energies at all TMs in the simulation results for the three complexes. At TM1 it was found to be substantial only in the KET simulation, consistent with the conformational changes we observed for TM1 in different systems (see above), and at TM5 it appeared to be relatively similar in all three complexes, but somewhat more pronounced in the 5-HT-bound structure; lastly, the residual exposure at TM6 is largest as well in the 5-HT trajectory, possibly due to the relatively straighter configuration of this helix in the 5-HT simulation (Figures 3–4). One possible mechanism to reduce the energy penalty for this residual exposure in the membrane-embedded receptor conformation produced by the binding of a particular ligand, is to bring together the TM domains where the residual exposure is largest. Therefore, we proposed [32] that this represents a membrane-determined energy drive for the association of the proteins in the membrane.

Consequently, our data in Table 2 of [32] suggests that if the hydrophobic mismatch is the driving force for receptor oligomerization, then the contact interfaces for oligomerization of the 5-HT_2A_R will be different in the complexes with 5-HT, LSD, or KET. According to this mechanism, ligands will not only regulate *the extent* of GPCR oligomerization, but will also influence *which TM domains* constitute the oligomerization interface. Thus, a comparison of residual surface area values at different TMs in 5-HT, LSD, and KET simulations implicates TM1, TM4 and TM5 as likely participants in the oligomerization interface of 5-HT_2A_R in complex with KET, TM4 and TM5 in the oligomerization interface of LSD-bound receptors, and TM5 (and possibly TM6, TM4 and TM2 as well) as the most likely participants in the oligomerization of 5-HT-bound serotonin receptor.

In addition, the results in Table 2 of [32] for the 5-HT and KET simulations imply that overall the inverse agonist KET will promote more extensive hydrophobic mismatch-driven oligomerization, since the residual surface area value summed over all TMs is about 90 Å^2^ higher for KET-bound 5-HT_2A_R than it is for 5-HT-bound receptor. This prediction is in excellent agreement with the experimental data on ligand-regulated oligomerization on β_2_AR [31], where in comparison to the agonist isoproterenol, the binding of an inverse agonist was suggested to promote tighter packing on β_2_AR protomers and/or to result in formation of higher-order oligomeric structures.

With regard to the validation of the ligand-dependent dynamic properties, it is important to note that similar residual exposure is observed in the two KET-bound simulations starting from very different initial conformations. Thus, the trend of large residual exposures at TM1, TM4, and TM5 of the KET system is also observed in the KET-substituted system (Table S3 in Text S1). Moreover, near the TMs where the hydrophobic mismatch is alleviated by the membrane remodeling (e.g., TM6), the membrane has similar thickness in both the KET and KET-substituted system (Figure S6 in Text S1).

## Discussion

The MD simulations of the 5-HT-, LSD- and KET-bound 5-HT_2A_R reported here provide the first molecular representation of the different effects that pharmacologically distinct ligands can have on the 5-HT_2A_R. The concepts of “functional selectivity” [49], [50] and “receptor bias” [51] are frequently being used to explain the increasingly common observation of differential responses elicited by different ligands from the same receptor (e.g., for 5-HT_2A_R see [4], [52]). However, no structural context had been identified for the distinct effects on the dynamics produced in the same GPCR by the binding of pharmacologically different ligands. Here we simulated the dynamics of the 5-HT_2A_R binding of such pharmacologically distinct ligands, and identified different effects on the SM/FMs of the receptor. These effects were shown to lead to different rearrangements that correspond to the different levels of activation known to be produced by these ligands. Notably, the differential effects were shown to be consonant with the pharmacological characterization of the three ligands as a full, partial and inverse agonist, respectively. To our knowledge, such inferences were obtained for the first time here from unbiased atomic MD simulations, but they are in line with the increasingly detailed experimental evidence about ligand-related functional selectivity [49], [50], [51], [53], [54], [55], [56], [57], [58], [59], [60], [61], [62], [63], [64], [65], [66], with the proposals of ligand-selective conformations in the 5-HT_2A_R [67] and the D_2_R [68], and with structural data indicating that GPCRs such as β_2_AR are stabilized in distinct conformational states by inverse, partial, or full agonists - respectively [12], [13].

In the current simulations, structural changes associated with SM/FM characteristics of an “activated state” of the 5-HT_2A_R appear in sub-microsecond trajectories. In contrast, experimentally determined GPCR activation timescales generally vary from microseconds (photoactivation of rhodopsin [69]) to seconds (β_2_AR in living cells [70]). We emphasize that the conclusions reached here do not require the simulations to have converged to an “active state structure” of the kind claimed for the constructs determined crystallographically. Indeed, a number of modes of activation proposed from experiment share this characteristic and can also be significantly faster [71], [72], [73]. But in general, there are many reasons for the time scale differences between our results and functional measurements. In particular, the simulated system is an idealized construct in that all interaction components are placed in optimal positions to be at or near their targets. Titratable groups are also assigned their final charge states, e.g., when the D3.49 and E6.30 are in the protonated form in some of the constructs. Interestingly, the specific protonation form does not determine whether the ionic lock is formed or not (see Figures 3–4, and Figure S5 in Text S1); rather, the determinant factor is seen from our results to be the nature of the dynamics induced by the binding of a specific ligand. But considering that inactive GPCR (β_2_AR) may be pre-coupled to G-protein Gs [31] and the protonation of E3.49 in rhodopsin (an activation step) depends on transducin [74], the degree of precoupling will likely play a role in the activation time. Moreover, the simulation conditions (such as pH, salt, lipid composition, and crowding) certainly do not mimic completely those surrounding the receptor in living cells (e.g., it is known that the highly flexible DHA chain of SDPC, included in the lipid mixture used here, facilitates GPCR activation [75]), and similar time-scale differences have been observed between computer simulations and experiments for other GPCRs [76], [77].

The response of the membrane environment to the different ligand-induced structural re-arrangements produces a reorganization of the membrane around the receptor. This is reflected in ***(i)-***the involvement of Chol in direct interactions with the protein [43], [78], that was shown here to affect the dynamics of the SM/FMs, and ***(ii)-***the membrane deformations around the TM bundle of a GPCR [48], [79], described here with the use of the CTMD method [32]. Because the different ligand-determined conformational changes in 5-HT_2A_R establish different patterns of local perturbations in membrane structure around the receptor complex, they were suggested to promote different ligand-dependent receptor oligomerization patterns through the hydrophobic mismatch between the TMs and the surrounding membrane [32]. This is supported by observations in the literature that: (*i*)- oligomeric associations of the dopamine D_2_R [27], 5-HT_2C_R [28], and the β_2_AR [31] is ligand-sensitive; and (*ii*)- GPCR self-assembly is regulated by the mismatch between the hydrophobic length of the TM segments of GPCRs and the hydrophobic thickness of the lipid bilayer, as suggested by both experimental results [80] and computational studies for rhodopsin [32], [48], [79]. Along these lines, the results presented here suggest that the dimerization interfaces of 5-HT_2A_R oligomers will be different for inverse agonist-, partial agonist-, or agonist-bound complexes, and moreover that the inverse agonist KET would promote more extensive 5-HT_2A_R oligomerization than the full agonist (5-HT). We note that these experimentally testable predictions regarding possible oligomerization interfaces were obtained by analyzing *monomeric* GPCRs in complex with different ligands, without the need to simulate the dimers or higher oligomers.

## Methods

### Construction of the simulated systems

Several model systems of the serotonin 5-HT_2A_R receptor were studied with all-atom MD simulations in explicit models of the hydrated lipid membrane environment. The 5-HT_2A_R was simulated in complex with three ligands known to exhibit different pharmacological efficacies: the full agonist 5-HT, the partial agonist LSD, and the inverse agonist KET (Figure 2A).

#### 5-HT_2A_R constructs

For the simulation of 5-HT bound 5-HT_2A_R, the protein was modified twice, very slightly, in regions distal to the binding site and the SM/FMs. The original receptor construct had a specific truncation of IL3 so that it consisted of 296 residues, from H1.28 to D5.57 and from R6.21 to K7.73, with an Ala–Ala linker between them (H1.28–D5.75–AA–R6.21–K7.73, where “–” denotes truncation). To match observations in crystallographic structures of several GPCRs [20], [25], [81], we thus added, at 112.5 ns, four residues to the IL3 (H1.28–L5.79–AA–S6.17–K7.73) in order to extend helical parts of TM5 and TM6, respectively, by two turns. The extension was done as follows: an average structure of the protein (including the ligand and palmitoyl derivative) was obtained from the trajectory between from 83.5 to 112.5 ns. The averaged structure was minimized first with constrained protein backbone and ligand heteroatoms followed by minimization without constraints. To enhance the flexibility of the truncated IL3, we extended the intracellular ends of TM5 and TM6 by 2 turns of helix each using Modeller [82], and selected the representative model by clustering the 100 models using either the extended TM5 or TM6. The loop between the extended TM5 and TM6 was refined using Modeller. The protein with extended TM5 and TM6 together with the ligand and palmitoyl chain was minimized first with protein backbone and 5-HT heteroatoms constrained, followed by complete minimization. The minimized complex was inserted in the lipid/water/ion environment from the snapshot at 112.5 ns to conserve interactions, after which the entire system was minimized and equilibrated with constraints on the protein backbone (velocities were reassigned in a random distribution based on the temperature). For the second extension, at 174.2 ns (i.e., 61.7 ns after extending TM5 and TM6), we added three more residues at the N-terminus (S1.25–L5.79–AA–S6.17–K7.73) to allow TM1 to reach beyond the lipid phosphate group region of the model membrane so as to avoid artificial interactions between the positive N-terminus and negative phosphate groups in membrane lipids. In addition, the N-terminus was acetylated and the C-terminus was N-Methylamidated to further avoid charge-charge interactions between termini and lipids. The simulations were then continued and the results reported here are from the 350 ns trajectory. Note that the initial homology model of 5-HT_2A_R includes an artificially open “ionic lock” between residues R3.50 and E6.30 due to the use of the β_2_ adrenergic receptor (β_2_AR) template in the homology modeling [83]. In the β_2_AR X-ray structure [45] the ionic lock is broken due to the co-crystallized lysozyme, but has been shown to consistently reform in MD simulations of inactive β_2_AR without the lysozyme [40].

The simulations of LSD-bound and KET-bound 5-HT_2A_R, started from the same conformation as for the 5-HT bound 5-HT_2A_R except that they included the extensions from the very beginning. In addition, to test whether KET, as an inverse agonist, is capable of reversing the conformation induced by the bound agonist 5-HT, we substituted 5-HT with KET in the activated 5-HT_2A_R structure obtained at the end of the 5-HT simulation, and restarted that simulation with KET for an additional 500 ns (termed “KET-substituted simulation”). The protocol for this switch of ligand was as follows: (i)-An average structure (protein+5HT+palmitoyl chain) was generated using the last 50 ns of 5-HT simulations, and then minimized; (ii) 5HT was substituted by KET so that the docking pose of KET (Figure 2D, left panel) is aligned with the minimized average structure using backbone atoms of binding site residues: D3.32, S3.36, S5.42 and S5.46. The complex (protein+KET+palmityol chain) was minimized by fixing the heteroatoms of KET and constraining backbone atoms of the protein; (iii)-The minimized complex was combined with the lipid/water/ion environment from a snapshot at 350 ns of the 5-HT simulation, to conserve the interaction between the protein and the environment. Lipid/water/ion was minimized and then equilibrated. Finally the whole system was equilibrated by reducing constraints on protein backbone atoms and KET heteroatoms. Velocities were reassigned based on the temperature.

Residues D3.49 and E6.30 were protonated in the 5-HT and LSD simulations (see also Discussion section, above), and deprotonated in the KET simulations (including KET-substituted simulation). We note that the protonation state of the E6.30 residue does not affect the state of the ionic lock, as we show in the separate simulation of KET-bound 5-HT_2A_R where E6.30 residue is protonated (see Figure S5 in Text S1).

In all simulations, C7.70 was palmitoylated by moving the coordinates of the palmitoyl chain (PALM) from PDB 2RH1 [45] onto the C7.70 of 5-HT_2A_R.

#### Loop structures determined from ab inito loop prediction

To enable full-scale 5-HT_2A_R simulations, we refined the loops in 5-HT_2A_R homology model described recently [83] using the Monte Carlo-Scaled Collective Variables *ab initio* method [84], [85]. For details see Methods and Table S2 in Text S1.

#### Initial placement of the ligands

Protonated 5-HT, LSD and KET were docked into 5-HT_2A_R using several docking protocols, including Autodock 4 [86], Simulated Annealing-Docking [87], Glide (Schrödinger Inc.), and IFD (Schrödinger Inc.). In Autodock, the GA-LS algorithm and a maximum number of 2.5×10^7^ evaluations were used. Simulated Annealing-Docking was carried out following a protocol previously established in our lab [87], [88] starting from a binding pose of 5-HT predicted by Autodock and consistent with experimental data. Glide [89] was carried out with and without H-bond constraints on D3.32 and/or S5.46. Applying H-bond constraints on S5.46 generated more docking poses that were consistent with the experimental data. IFD [90] was carried out starting either from scratch or from Glide docking poses that were consistent with experimental data. Other docking parameters were set to default values.

These procedures generated docking poses consistent with experimental data in the literature [2], [6], [7], [91], [92] (Figure 1A–D). In particular, for KET, IFD produced a cluster of docking poses in which the amines of the ligands interacted with D3.32 and S5.46, and its quinazoline ring immersed deep into the binding pocket close to W6.48. The binding site remained almost unchanged except that F6.52 rotated to avoid steric clashes with KET (Figure 1D). In this docking pose, which was used in the simulations, KET was in direct contact with the aromatic cluster (F5.47, F6.44, W6.48, F6.51 and F6.52) by forming an edge-to-face interaction with W6.48.

#### Internal waters

X-ray structures of several GPCRs show water networks around the toggle switch W6.48 and the NPxxY motif [45], [93], [94], [95], and these are hypothesized to be important for receptor activation [96]. Internal waters were therefore introduced by solvating the 5-HT_2A_R with Grand-Canonical Ensemble simulations using the Monte Carlo program MMC [97]. The procedure placed waters around W6.48 and the NPxxY motif consistent with the X-ray structures of cognate GPCRs.

#### Lipid membrane composition and protein-membrane complex preparation

The 5-HT_2A_R-ligand complexes were embedded in identical mixed and hydrated 7∶7∶6 1-stearoyl-2-docosa-hexaenoyl-sn-Glycero-3-phosphocholine (SDPC)/phosphatidylcholine (POPC)/Chol membranes. The choice of the lipid mixture was dictated by several factors: (*i*)-Chol is known to be important for modulating ligand binding, G-protein binding and activation of serotonin receptors [44], and has even been found in complex with the X-ray structures of amine GPCRs elucidated recently; (*ii*)-POPC represents a typical phospholipid component of the bilayer membrane, with one saturated and one mono-unsaturated acyl chain; and (*iii*)-SDPC lipid has been implicated specifically in the function of various GPCRs [98] and is abundant in neuronal tissues. In addition, the use of this lipid composition enables a comparison of Chol dynamics around 5-HT_2A_R with observations from earlier MD studies of rhodopsin in somewhat different Chol-containing mixed membranes [43].

The lipid bilayer model was generated using VMD [99] to construct first a 120 Å×120 Å (in the x-y plane) hydrated POPC membrane patch consisting of 406 lipids; then, half of the POPC lipids were transformed to SDPC by translating corresponding atoms, i.e., from the POPC headgroups to PCGL, from the 16∶0 *sn-1* chain to STEA, and from the 18∶1 *sn-2* tail to DHA (missing atoms were built using internal coordinates in the all-atom CHARMM27 force field [100] with CHARMM31b1 [101]). To reduce steric clashes between POPC and SDPC molecules, we made use of the relatively straight DHA chains from the equilibrated SDPC membrane bilayer (http://www.lipid.wabash.edu/), and replaced all the DHA chains in the current membrane patch with the straight DHAs. The 5-HT-, LSD-, or KET-bound 5-HT_2A_R were inserted into the lipid matrix by aligning the backbone of its seven most conserved residues (one in each TM, see [18]) with those of rhodopsin immersed in a POPC membrane [22]. Lipids within 0.8 Å of the protein and PALM were then removed leaving 354 lipids in total. 26 SDPC and 26 POPC in each leaflet were randomly replaced with Chol (PDB 3D4S [46]), by fitting Chol's C4, C5 and C6 atoms to STEA's C5, C6 and C7 or POPC's C35, C36 and C37. Chol positions were then refined by lateral translation to avoid clashes with other Chol, SDPC or POPC lipids. Finally, the systems were solvated with TIP3 water and 0.15 M NaCl salt. The final simulated systems consisted of 125 SDPC, 125 POPC, 104 Chol and 20–22K water molecules resulting in a total of 106–114K atoms.

### Force-fields and MD simulations

The parameters for 5-HT were taken from an earlier study [7]. For LSD and KET, the results of geometry optimization and electrostatic potentials obtained from quantum mechanical calculations with the Gaussian program (Gaussian, Inc., Wallingford, CT) were used as input to the Restrained-ElectroStatic-Potential fit method [102] implemented in Antechamber [103] to derive charges. CHARMM topology and parameter files were then prepared with Antechamber using Restrained-ElectroStatic-Potential charges and GAFF force field. Force field parameter files for 5-HT, LSD and KET are included in Text S1. For protein, PALM, lipids etc., the all-atom CHARMM27 force field with CMAP corrections [100] was utilized (this approach is similar to a procedure used successfully in previous studies [104], [105]).

All MD simulations were performed with the NAnoscale Molecular Dynamics (NAMD) suite [106]. As established in similar studies in the lab (e.g., see [107]), the simulations were conducted under constant temperature and pressure conditions with anisotropic pressure coupling, and utilized PME for long-range electrostatics [108]. The Nose-Hoover Langevin piston method [106] was used to control the target pressure with the LangevinPistonPeriod set to 100 fs and LangevinPistonDecay set to 50 fs. All MD simulations were performed with rigidBonds allowing 2 fs time step.

All the simulated systems were equilibrated following a procedure described recently [109]. According to this protocol, the 5-HT_2A_R backbones and the heavy atoms of the ligands were initially fixed and then harmonically constrained, and water was prevented from penetrating the protein-lipid interface. Constraints were released gradually in four 300 ps-step MD simulations with decreasing force constants of 1, 0.5, 0.1 and 0.01 kcal/(mol·Å^2^), respectively. Following this equilibration phase, all three GPCR-membrane complexes were simulated for 350 ns.

The stability of the simulated complexes was monitored from the backbone RMSDs of the TMs in 5-HT_2A_R using the following definitions for TMs: L1.29–L1.59, A2.38–Y2.67, L3.24–N3.56, S4.38–V4.62, D5.35–K5.67, N6.29–I6.60, G7.32–F7.56 and K7.58–I7.68. As illustrated in Figure 1E, after initial equilibration, the RMSDs in all the three systems were stable and fluctuated around or below 2 Å. In all three simulations the ligands maintained key interactions with the receptor (Figure 1B–E), consistent with previous experimental data [2], [6], [7], [91], [92].

### Analysis of MD trajectories

To quantify the changes in protein structure produced by the simulations we used various analysis tools. Analysis of protein structural data was carried out with Ptraj in AMBER 9 [110] and other tools discussed below. To quantify helix distortion parameters in the simulations, we used the Prokink package [111] implemented in Simulaid [112]. The correlation analysis on the time-dependent data of different variables, such as helix bend angles, face-shifts, as well as Chol-protein distances, was conducted following the procedure described in [43]. Briefly, the correlation analysis was carried out on two separate sets of dynamic variables. In the first, we followed the time-sequence of *m* = 8 selected variables that included proline kink and face-shift angles in TM6 and TM7, the minimum distances between the Chol at the EC end of TM6 and the residues on TM6 (I6.53, M6.57, I6.60, C6.61). In the second set, *m* = 12 dynamic variables were selected that included proline kink and face-shift angles in TM6 and TM7, the minimum distances between the Chol at the IC end of TM6–7 and the residues on TM6 and TM7 (K6.35, I6.39, F6.42, V6.46, L7.44, V7.48, V7.52, L7.55, F7.56).

For each set, we first studied pair-wise correlations between different variables, and constructed the matrix of coefficients of determination, *R* ^2^ (Figure 7D of the main text) using Spearman's rank correlation test (see for instance Ref. [113]). In this method, given *N*_frames_ pairs of observations, (*x_i_*, *y_i_*), first the *x_i_* and *y_i_* values separately are assigned a rank, and then the corresponding difference, *d_i_* between the *x_i_* and *y_i_* ranks is found for each pair. The *R* ^2^ is then defined as:
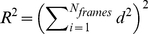
(1)Because it uses rankings, Spearman's method eliminates the sensitivity of the correlation test to the function linking the pairs of values and thus is preferred over parametric tests when no *a priori* knowledge exists on the functional relationship between *x_i_* and *y_i_* pairs.

### Combined Essential Dynamics (Comb-ED) analysis

To compare the conformational spaces of 5-HT_2A_R stabilized by the different ligands (i.e., 5-HT, LSD and KET), a Combined Essential Dynamics analysis [42], [114] was performed on C_α_ atoms of the protein using Gromacs 3.3 [115]. Essential dynamics analysis separates the configurational space into an essential subspace with a few degrees of freedom which describe overall motions of the protein that are likely to be relevant to its function, and a physically constrained subspace describing local fluctuations. The method is based on the diagonalization of the covariance matrix of atomic fluctuations defined by:
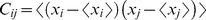
(2)where *x_i_* are the three Cartesian coordinates of the carbon atoms C_α_ of the molecule under study, and the angular brackets denote averages over an ensemble of configurations and over the simulation time. The diagonalization of Eq. (3) yields eigenvectors that describe the directions of correlated positional changes in the molecule, whereas the eigenvalues indicate the total mean square fluctuation along these directions.

In the Comb-ED, the covariance matrix is calculated for two or more concatenated trajectories, which are fitted on the same reference structure. Given this construct, the eigenvectors resulting from Comb-ED do not represent the essential degrees of motion of the molecules, but rather reveal differences and/or similarities in the dynamical and structural characteristics of the compared simulated structures. To identify structural differences between 5-HT_2A_R stabilized by the three ligands, Comb-ED analysis was performed on 3 concatenated trajectories obtained by combining the trajectories for the pairs 5-HT-LSD, 5-HT-KET, and LSD-KET, each for the last 100 ns, respectively.

#### Analysis of membrane deformations and the residual mismatch

The properties of the membranes were analyzed from the simulation trajectories using the recently described CTMD method [32]. Briefly, to quantify membrane deformations in the simulations and the hydrophobic mismatch energies, we calculated the time-averaged hydrophobic thickness profile of the membrane surrounding 5-HT_2A_R in all trajectories and used solvent accessible surface area calculations to calculate the energy of the residual mismatch which exposes TM residues participating in unfavorable interfacial hydrophobic/hydrophilic interactions. To identify these residues, we determined if the TM is thicker or thinner than the surrounding membrane by comparing the hydrophobic thicknesses of the TM domains (using the following TM definitions (given in the Ballesteros-Weinstein generic numbering [18]): 1.29–1.59 (TM1), 2.38–2.67 (TM2), 3.24–3.53 (TM3), 4.39–4.63 (TM4), 5.38–5.63 (TM5), 6.33–6.59 (TM6), 7.30–7.56 (TM7)) to the local membrane thickness *d_memb_* calculated from the membrane sectors corresponding to each TM, as described in [32].

## Supporting Information

Text S1 **Including supplemental methods, Figures S1, S2, S3, S4, S5, S6, Tables S1, S2, S3, Topology and parameter files for 5HT, LSD and KET.**
(DOC)
