# Supplementary material for: Ligand-Dependent Conformations and Dynamics of the Serotonin 5-HT2A Receptor Determine Its Activation and Membrane-Driven Oligomerization Properties
Source: PLoS Comput Biol. 2012 Apr 19;8(4):e1002473. doi: 10.1371/journal.pcbi.1002473 (PMC3330085; doi:10.1371/journal.pcbi.1002473)
Supplement: Text S1 — Including supplemental methods, Figures S1, S2, S3, S4, S5, S6, Tables S1, S2, S3, Topology and parameter files for 5HT, LSD and KET. (DOC) [file pcbi.1002473.s001.doc]

**TEXT S1**

Ligand-dependent conformations and dynamics of the serotonin 5-HT_2A_ receptor determine its activation and membrane-driven oligomerization properties

Jufang Shan, George Khelashvili, Sayan Mondal, Ernest L. Mehler, Harel Weinstein

**Supplemental Methods**

**Ab initio loop structure prediction with the Monte Carlo-Scaled Collective Variables method (MC-SCV)**

Two rounds of loop calculations were carried out. First, loops were modeled separately and subjected to a protocol consisting of four steps of MC-SCV calculations (for details see ref. [1,2,3,4]). Step-1 uses MC-Simulated Annealing to generate an ensemble of folded loops from a fully extended polypeptide chain followed by 3 steps using MC-SCV; Step-2 performs opening-closing of the loops at 310 K in the field of protein and continuum solvent to relax the fold; Step-3 selects low energy loop conformations from Step-2, replicates them, opens, heats and closes each ensemble of loops at 1210 K, which facilitates crossing energy barriers and finding the native ensembles; and Step-4 selects low energy loops from Step-3, replicates them, and opens-closes each ensemble at 310K. The ensembles in Step-4 are ranked based on an Helmholtz-like free energy

$\Delta A=E_{\min}-RT\ln Q$ (1)

Where

$Q=\sum_{i=1}^{M} \exp[-\frac{E_{i}-E_{\min}}{RT}]$ (2)

with *M* denoting the number of replicas used in the MC calculation, and *E_min_* and *E_i_* representing the minimum energy of the given ensemble, and the energy of the *i*'th conformation in the distribution, respectively. The ensemble with the lowest Δ*A* is assumed to be the best representative of the native ensemble.

Extracellular loop 1 (EL1) and intracellular loop 1 (IL1) were not included in the first round, because they share similar (short) lengths with EL1 and IL1 in rhodospin [5] and β_2_AR [6]. In addition, these loops in rhodopsin and β_2_AR share common structures as well (RMSD < 1.1Å). Taken together, EL1 and IL1 in the three GPCRs, 5-HT_2A_R, rhodopsin and β_2_AR, should be structurally similar.

For EL2, with a conserved disulfide bridge between its Cys227 and C3.25 (Ballesteros-Weinstein numbering [7]) a special protocol was developed that allowed the loop sequence to be split into two shorter segments. The first segment (EL2a) ran from the loop's N-terminus to the disulfide bridged Cys227 while the second segment (EL2b) ran from Cys227 to the loop's C-terminus peptide. Segment EL2b was calculated first and then EL2a was predicted in the presence of EL2b.

In the second round of calculations, all the loops were assembled and refined one by one in the order of IL2, IL3, IL1, and EL2, EL3, EL1. For EL1 and IL1, previous built homology models [1] were used (see below) as initial structures. For IL2, a previously built helical structure [1] was used. In this round, the loops were only subjected to Step-2 (open-close at 310K) in order to relax them in the field of protein, solvent and other loops. EL2a and EL2b were opened and closed simultaneously using a special algorithm designed for the EL2 [2]. All the loops produced ensembles with small energy and RMSD spreads, indicating that they belonged to the native ensembles [3].

The sequences of the predicted loops are given in Supplemental Table S2. Note, that since the original IL3 is much longer compared to the other loops, and therefore difficult to investigate with *ab initio* methods, we truncated IL3 but still kept residues critical for G-protein coupling, arrestin binding and downstream signaling in cognate GPCRs [8,9,10,11]. GPCRs with such truncated IL3 or chimeric GPCRs consisting of TM1-5 and TM6-7 (without IL3) segments taken from different GPCRs are known to maintain ligand-related functional elements [12,13,14,15,16,17].

We note that, except for short and conserved loop segments EL1, IL1 and EL2b, the predicted loops are different from those of β_2_AR, although the transmembrane helices (TMs) from the homology model are similar to those in β_2_AR (with a backbone RMSD of 0.6 Å based on the main chain superposition of the seven I.50 residues). EL2a in 5-HT_2A_R is shorter than that in β_2_AR, nonetheless it has some helical contents although shorter than the two-turn helix in β_2_AR. Our previous comparative study of the structural properties of IL2 in wild type β_1_AR, β_2_AR and 5-HT_2A_R GPCRs, as well as in the corresponding P5.37A mutants showed the existence of at least two major conformational families for IL2 in the cognate GPCRs. Furthermore, we found that it is most likely the helical conformation that binds to β-arrestin and therefore is a part of the active form of these GPCRs [1]. Thus, in our simulations of 5-HT_2A_R with 5HT and LSD the IL2 conformation was helical similar to that in β_1_AR [18]. Since 5-HT_2A_R has some basal activity in the presence of KET, a helical IL2 was also used in the KET simulation.

**Preferred Cholesterol binding sites around 5-HT_2A_R and rhodopsin**

To determine preferred cholesterol (Chol) binding sites around 5-HT_2A_R, for each simulation, we first identified the Chol molecules in contact with the receptor as those within 2.5 Å of the protein at any time-point during the entire 350 ns simulations. In addition, we also tracked those Chol that were within 10 Å radial shell around 5-HT_2A_R during the simulations. Note that the latter group of Chol molecules are not necessarily in direct contact with the GPCR. The Chol molecules were then classified according to the percentage of time they spend in contact with the protein (within 2.5 Å of the GPCR), as well as the percentage of time they reside within 10 Å radial shell around 5-HT_2A_R. At the same time, the information was gathered about the protein residues nearest to the Chol. From the location of these residues we identified the TM regions participating in interactions with Chol.

The information gathered from this analysis is consolidated in the Table S1. Note that in addition to the three 5-HT_2A_R sets of simulations, we performed a 250 ns simulation of rhodopsin in the same SDPC/POPC/Chol membrane. This new simulation was conducted in order to compare Chol dynamics around different GPCRs, as well as in different lipid membrane (IBM simulations, see [19,20]), with the ultimate goal of constructing a generalizable model of Chol involvement in GPCR function. *The entries in Table S1 are colored and labeled according to our Chol ranking described above (see captions for details).*

**Supplemental Figures**

**
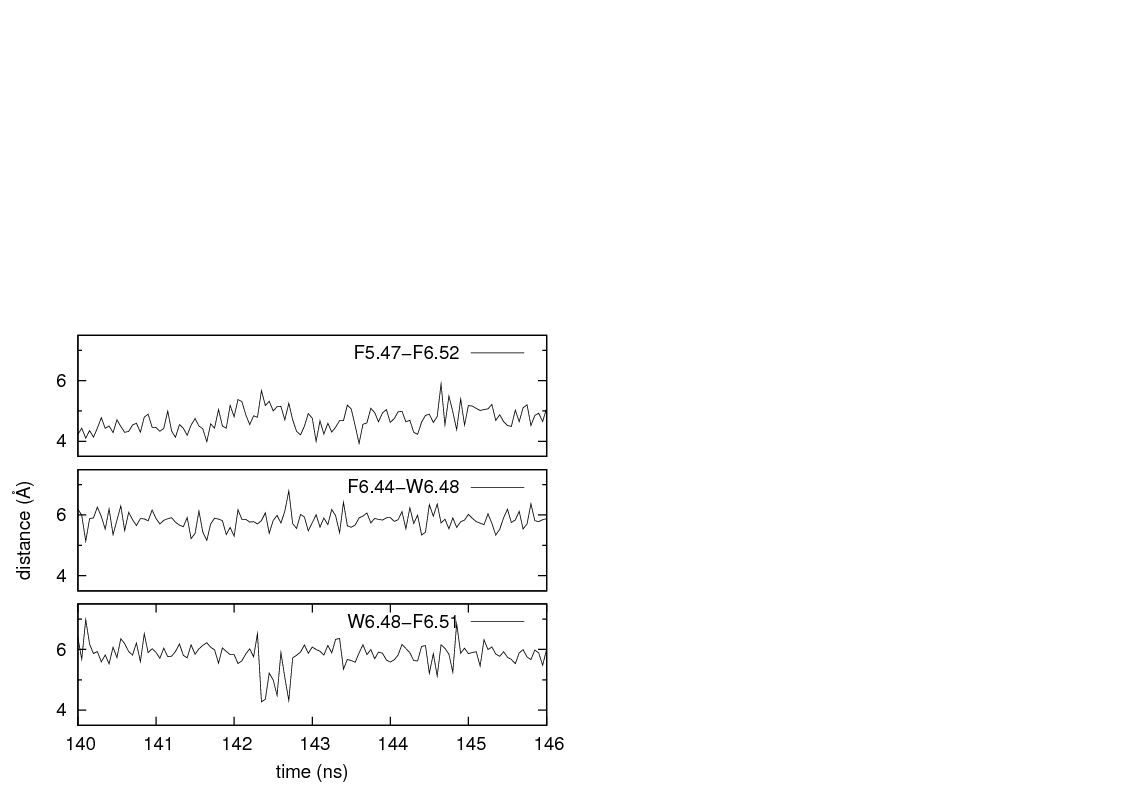
**

**Figure S1.** Distances between the centers of aromatic-residue rings in the 5-HT_2A_R simulation. Some residues in the aromatic cluster form pi-pi interaction. Some of these interactions persist through the simulations (F5.47–F6.52) while others evolve with the flipping of W6.48, e.g., W6.48–F6.51.

**
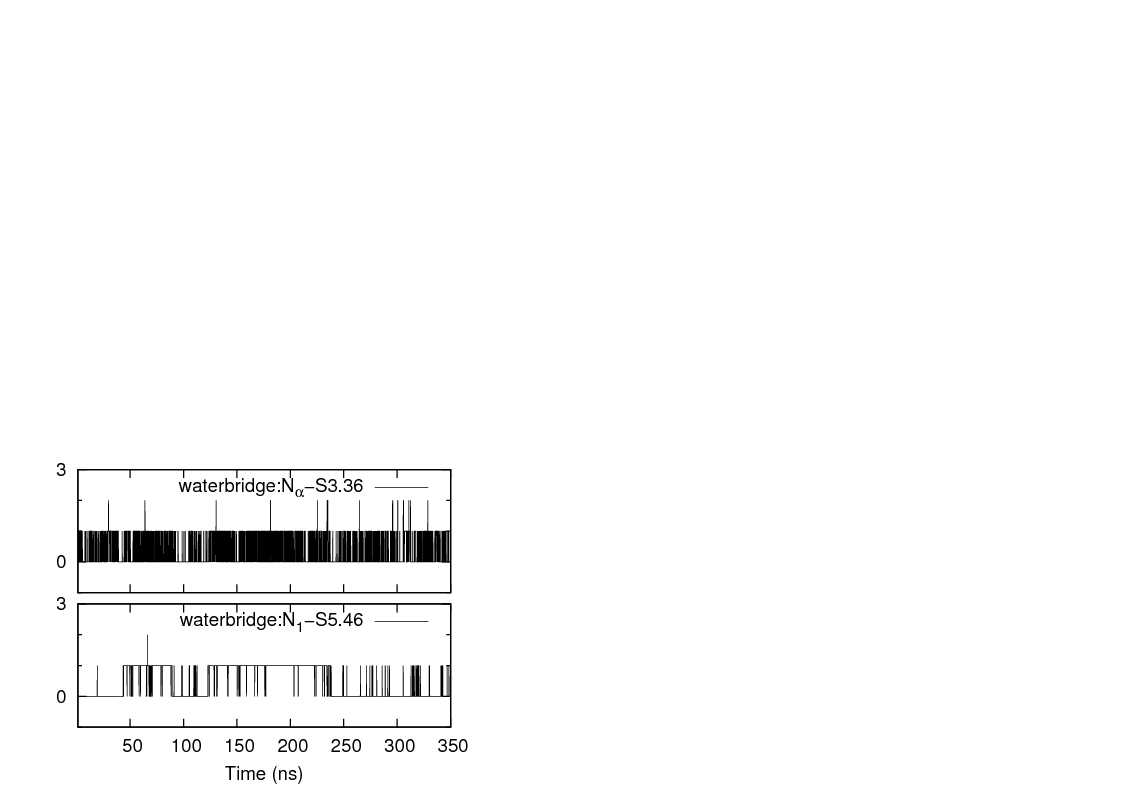
**

**Figure S2.** Number of water molecules between N_α_ on 5-HT and S3.36 of 5-HT_2A_R (top panel), and between N_1_ on 5-HT and S5.46 of 5-HT_2A_R (bottom panel). Waters are counted if their oxygen are within 3.5Å of both the nitrogen on 5-HT and hydroxyl oxygen of 5-HT_2A_R. Data were collected every 100 ps.

**Figure S3.** Comb-ED analysis performed on a concatenated trajectory composed of four separate simulations: 5-HT, LSD, KET and KET-substituted. The four simulations are in four distinct clusters and only the TM residues are followed. The largest variation among these four clusters is along the first eigenvector; 5HT and LSD are close to each other along this coordinate, but far away from Ket, while the Ket-substitution has already moved away from 5HT and LSD, and closer to KET. This is consistent with our observation, from monitoring RMSD and SM/FMs, that the substituted 5-HT-to-KET system is exhibiting dynamics similar to those produced by the inverse agonist.

**
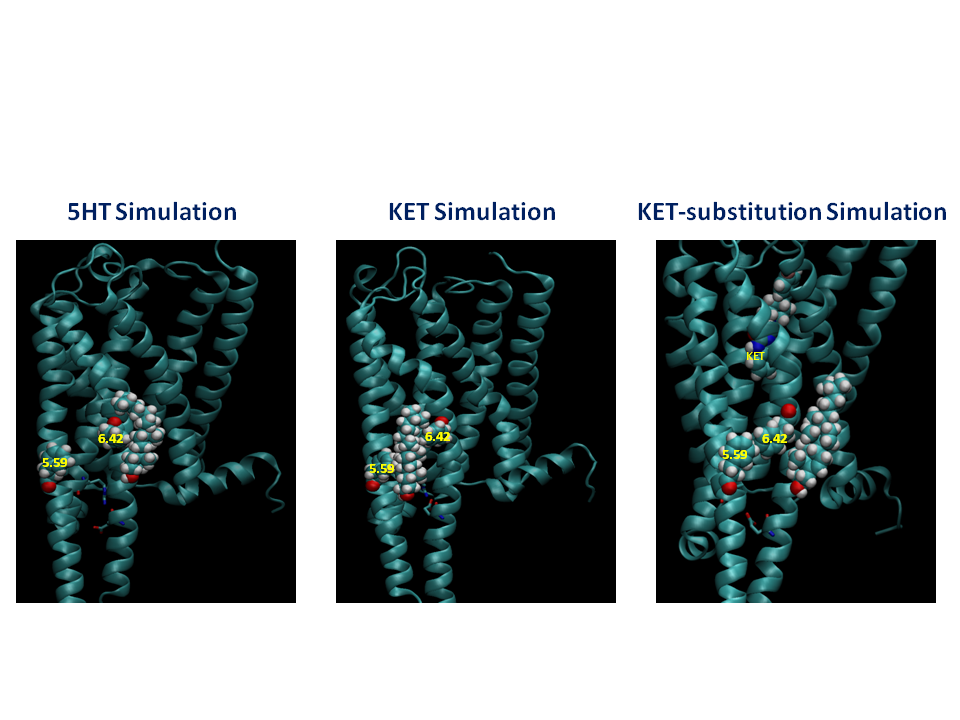
**

**Figure S4.** Snapshots of 5-HT_2A_R conformations (shown in cartoon) stabilized by 5HT (left), KET (middle), and by KET substituted into 5HT structure (right). F5.59 and F6.42 residues on TM5 and TM6 respectively are in spheres. Cholesterol neighboring TM6 is in spheres. The ionic lock pair R3.50–E6.30 is depicted in licorice.

**Figure S5.** Distance between the C-alpha atoms of E6.30 and R3.50 residues in KET-bound 5HT_2A_R simulations with protonated E6.30. The plot includes initial equilibration phase (shaded region), followed by ~55ns of unbiased simulations.

**
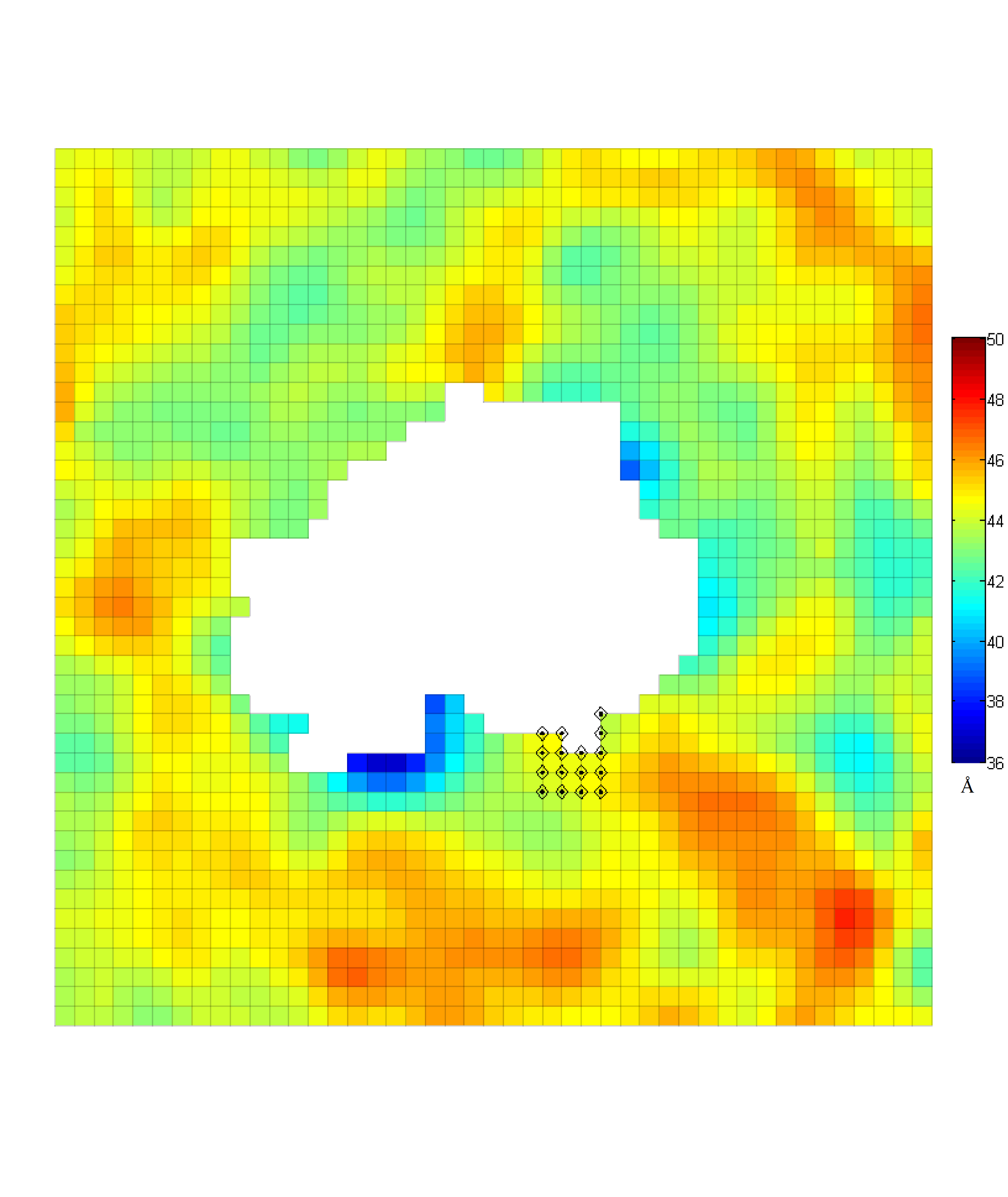
**

**Figure S6.** Hydrophobic thickness profile of the simulated membrane around 5-HT_2A_R in complex with KET that substituted 5HT. Figure was prepared as Figure 8 and the membrane region near TM6 is highlighted.

**Supplemental Tables**

**Table S1. Preferred Cholesterol binding sites around 5-HT_2A_**R and rhodopsin in mixed SDPC/POPC/Chol membranes

|  | 5-HT_2A_R | | | | | |  | rhodospin | |
| --- | --- | --- | --- | --- | --- | --- | --- | --- | --- |
|  | 5-HT | | LSD | | KET | |  | 11-cis-retinol | |
| chol | Inter.*^a^* | Region *^b^* | Inter. *^a^* | Region *^b^* | Inter. *^a^* | Region *^b^* |  | Inter. *^a^* | Region *^b^* |
| 1 | - |  | - |  | stays | EC/6 |  |  |  |
| 2 | transient |  | stays |  | stays | EC/6 |  | stays | EC/5-6 |
| 3 | stays |  | stays | EC/6 | stays | EC/6 |  | stays | EC/5-6 |
| 4 | stays | EC/1 | - |  | stays |  |  | stays | EC/5-6 |
| 5 | stays |  | stays | EC/1 | - |  |  | stays | - |
| 6 | stays | EC/1-2 | stays | EC/1-2 | stays | EC/1 |  | stays | EC/5-6 |
| 7 | stays |  | - |  | - |  |  | - |  |
| 8 | out |  | - |  | transient |  |  | stays | EC/5-6 |
| 9 | transient |  | - |  | stays | EC/1 |  | transient |  |
| 10 | stays | EC/2-3 | stays | EC/2-3 | stays | EC/2 |  | stays | EC/1-2-3 |
| 11 | stays | EC/3-4 | stays | EC/3-4 | stays | EC/4 |  | stays | EC/1-2-3 |
| 12 | stays | EC/2-3 | - |  | - |  |  |  |  |
| 13 | stays | EC/3 | transient |  | transient | EC/4 |  | out |  |
| 14 | - |  | - |  | - |  |  | transient |  |
| 15 | stays |  | stays | EC/5 | - |  |  | - |  |
| 16 | stays |  | - |  | - |  |  | transient | EC/6-7 |
| 17 | stays | IC/4 | - |  | - |  |  | - |  |
| 18 | stays |  | stays | IC/5 | stays | IC/5-6 |  | stays | IC5 |
| 19 | out |  | - |  | - |  |  | stays | IC/7-palm |
| 20 | stays | IC/6-7 | stays | IC/6 | - |  |  | stays | IC/6-7 |
| 21 | stays | IC/7-8 | stays | IC/1-7-8 | stays | IC/1-7 |  | stays | IC/6-7 |
| 22 | out |  | stays | IC/1 | stays |  |  |  |  |
| 23 | stays | IC/3-5 | stays |  | stays | IC/3-4-5 |  | transient |  |
| 24 | stays | IC/4 | stays | IC/4 | stays | IC/4 |  | stays | IC124 |
| 25 | stays |  | - |  | - |  |  | - |  |
| 26 | stays | IC/1-2-4 | stays | IC/2-4 | stays | IC/1-2-4 |  | stays | IC124 |
| 27 | stays |  | - |  | - |  |  | transient |  |
| 28 | transient |  | - |  | stays | IC/5 |  | - |  |

*^a^* Colors represent the extent of direct Chol-GPCR interactions: red, the Chol interacts (minimum distance < 2.5 Å) with the protein more than 75% of the time; green, between 25 and 75% of the time; grey, less than 25%. Labels (stays, in, out, transient) mean the percentage of time Chol were found within 10 Å shell around the GPCR: “stays”, the Chol is in the shell more than 80% of the time; “in”, in the shell between 20% and 80% of the time, *entering the shell* at some point of the trajectory and *staying inside the shell* till the end; “out”, in the shell between 20% and 80% of the time, *leaving the shell* at certain time point and *staying outside the shell* till the end; “transient”, in the shell between 20% and 80% of the time; “-”, stays in the shell less than 20% of the time.

**Table S2. Sequence for *ab initio*** loop prediction

| Loop | Sequence *^a^* |
| --- | --- |
| IL1 | 1.58SLEKKLQNAT2.39 |
| EL1 | 2.66LYGYRWPLPSK3.23 |
| IL2 | 3.54*I*QNPIHHSRFNS*RT*4.40 |
| EL2 | 4.62FGLQDDSKVFKEGSCLLAD5.35 |
| IL3 | 5.68EATLCVSD5.75--AA--6.21RRTMQSISNE6.30 |
| EL3 | 6.61CKESCNEDVIG7.32 |

*^a^* underlined are helical residues at the ends of corresponding TMs; italicized are residues only included in the second round of MC calculation; “--” designates truncations.

**Table S3. Residual Exposures (SA_res_) in the KET-substituted system**

| TM | SA_res_ (Å^2^) |
| --- | --- |
| 1 | 95 |
| 2 | 0 |
| 3 | N.D.* |
| 4 | 96 |
| 5 | 85 |
| 6 | 0 |
| 7 | 3.6 |

* N.D., Not Determined

**Topology and parameter files for 5HT, LSD and KET**

***Topology file for 5HT***

RESIDUE 5HT 1.0 ! serotonine, protonated.

GROUP

ATOM C1 CT2 -0.217

ATOM H11 HA 0.267 ! H51

ATOM H12 HA 0.272 ! H1 |

GROUP ! | H11 H21 C5 H61

ATOM C2 CT2 -0.483 ! | | | / \ /

ATOM H21 HA 0.252 ! H2--N1---C1---C2---C3-----C4 C6--O1

ATOM H22 HA 0.253 ! | | | | | |

GROUP ! | H12 H22 C10 C9 C7--H71

ATOM C3 CY -0.181 ! H3 / \ / \ /

ATOM C4 CPT -0.079 ! H01 N2 C8

ATOM C9 CPT 0.154 ! | |

ATOM N2 NY -0.623 ! H91 H81

ATOM H91 H 0.456

ATOM C10 CA 0.052

ATOM H01 HP 0.247

GROUP

ATOM C5 CA -0.296

ATOM H51 HP 0.241

GROUP

ATOM C6 CA 0.356

ATOM O1 OH1 -0.765

ATOM H61 H 0.508

GROUP

ATOM C7 CA -0.307

ATOM H71 HP 0.252

GROUP

ATOM C8 CA -0.221

ATOM H81 HP 0.257

GROUP

ATOM N1 NH3 -0.840

ATOM H1 HC 0.482

ATOM H2 HC 0.483

ATOM H3 HC 0.480

BOND C1 H11 C1 H12 C1 N1 N1 H1

BOND C1 C2 N1 H2 N1 H3

BOND C2 H21 C2 H22 C3 C4 C10 H01

BOND C2 C3 C3 C10 C4 C5 C4 C9

BOND C5 H51 C5 C6 C6 C7 C6 O1

BOND O1 H61 C7 C8 C7 H71 C8 C9

BOND C8 H81 C9 N2 N2 H91 N2 C10

DONO H1 N1

DONO H2 N1

DONO H3 N1

DONO H91 N2

DONO H61 O1

ACCE O1

! Internal coordinate definitions

IC H1 N1 C1 C2 0.0000 0.00 60.00 0.00 0.0000

IC N1 C1 C2 C3 0.0000 0.00 187.00 0.00 0.0000

IC C1 C2 C3 C10 0.0000 0.00 253.50 0.00 0.0000

IC H61 O1 C6 C5 0.0000 0.00 180.00 0.00 0.0000

IC C4 C10 *C3 C2 0.0000 0.00 180.00 0.00 0.0000

IC C9 C4 C3 C10 0.0000 0.00 0.00 0.00 0.0000

IC C3 C4 C9 N2 0.0000 0.00 0.00 0.00 0.0000

IC C9 C3 *C4 C5 0.0000 0.00 180.00 0.00 0.0000

IC C9 C4 C5 C6 0.0000 0.00 0.00 0.00 0.0000

IC C4 C5 C6 C7 0.0000 0.00 0.00 0.00 0.0000

IC C5 C6 C7 C8 0.0000 0.00 0.00 0.00 0.0000

IC C4 C6 *C5 H51 0.0000 0.00 180.00 0.00 0.0000

IC C7 C5 *C6 O1 0.0000 0.00 180.00 0.00 0.0000

IC C6 C8 *C7 H71 0.0000 0.00 180.00 0.00 0.0000

IC C9 C7 *C8 H81 0.0000 0.00 180.00 0.00 0.0000

IC C10 C9 *N2 H91 0.0000 0.00 180.00 0.00 0.0000

IC N2 C3 *C10 H01 0.0000 0.00 180.00 0.00 0.0000

IC C1 C3 *C2 H21 0.0000 0.00 240.00 0.00 0.0000

IC C1 C3 *C2 H22 0.0000 0.00 120.00 0.00 0.0000

IC N1 C2 *C1 H11 0.0000 0.00 118.00 0.00 0.0000

IC N1 C2 *C1 H12 0.0000 0.00 -118.00 0.00 0.0000

IC H1 C1 *N1 H2 0.0000 0.00 120.00 0.00 0.0000

IC H1 C1 *N1 H3 0.0000 0.00 240.00 0.00 0.0000

IC C7 C8 C9 C4 0.0000 0.00 0.00 0.00 0.0000

IC H81 C8 C9 C4 0.0000 0.00 180.00 0.00 0.0000

IC C7 C8 C9 N2 0.0000 0.00 180.00 0.00 0.0000

IC C8 C9 C4 C5 0.0000 0.00 0.00 0.00 0.0000

IC N2 C9 C4 C5 0.0000 0.00 180.00 0.00 0.0000

IC C8 C9 C4 C3 0.0000 0.00 180.00 0.00 0.0000

IC C4 C3 C10 N2 0.0000 0.00 0.00 0.00 0.0000

IC C3 C10 N2 C9 0.0000 0.00 0.00 0.00 0.0000

IC H01 C10 N2 C9 0.0000 0.00 180.00 0.00 0.0000

IC C3 C10 N2 H91 0.0000 0.00 180.00 0.00 0.0000

IC C10 N2 C9 C8 0.0000 0.00 180.00 0.00 0.0000

IC H91 N2 C9 C8 0.0000 0.00 0.00 0.00 0.0000

IC C10 N2 C9 C4 0.0000 0.00 0.00 0.00 0.0000

IC C3 C4 C5 C6 0.0000 0.00 180.00 0.00 0.0000

IC C9 C4 C5 H51 0.0000 0.00 180.00 0.00 0.0000

IC H51 C5 C6 C7 0.0000 0.00 180.00 0.00 0.0000

IC C4 C5 C6 O1 0.0000 0.00 180.00 0.00 0.0000

IC O1 C6 C7 C8 0.0000 0.00 180.00 0.00 0.0000

IC O1 C6 C7 H71 0.0000 0.00 0.00 0.00 0.0000

IC C5 C4 C3 C2 0.0000 0.00 0.00 0.00 0.0000

IC C9 C4 C3 C2 0.0000 0.00 180.00 0.00 0.0000

IC N2 C10 C3 C2 0.0000 0.00 180.00 0.00 0.0000

IC H21 C2 C3 C10 0.0000 0.00 15.00 0.00 0.0000

IC H22 C2 C3 C10 0.0000 0.00 133.00 0.00 0.0000

***Parameter file for 5-HT***

ANGLES

CT2 CT2 CY 51.800 107.5000 ! from CT1 CT2 CA (PAR27)

DIHEDRALS

CT2 CT2 CY CA 0.2300 2 180.00 ! from CT1 CT2 CY CA (PAR27)

CT2 CT2 CY CPT 0.2300 2 180.00 ! from CT1 CT2 CY CPT (PAR27)

OH1 CA CA CPT 3.1000 2 180.00 ! from OH1 CA CA CA (PAR27)

***Topology file for LSD***

* Topology File.

*

99 1

MASS 1 lna 14.010000

MASS 2 lcc 12.010000

MASS 3 lcd 12.010000

MASS 4 lc3 12.010000

MASS 5 ln4 14.010000

MASS 6 lc2 12.010000

MASS 7 lce 12.010000

MASS 8 lca 12.010000

MASS 9 lc 12.010000

MASS 10 lo 16.000000

MASS 11 ln 14.010000

MASS 12 lhn 1.008000

MASS 13 lh4 1.008000

MASS 14 lhc 1.008000

MASS 15 lhx 1.008000

MASS 16 lha 1.008000

MASS 17 lh1 1.008000

RESI LSD 1.000

GROUP

ATOM N1 lna -0.441808

ATOM C1 lcc -0.146181

ATOM C2 lcd -0.062140

ATOM C3 lc3 -0.088815

ATOM C4 lc3 -0.043140

ATOM N2 ln4 0.054172

ATOM C5 lc3 -0.637333

ATOM C6 lc3 0.037135

ATOM C7 lc2 -0.309572

ATOM C8 lce 0.105239

ATOM C9 lca -0.054047

ATOM C10 lca -0.212485

ATOM C11 lca -0.128160

ATOM C12 lca -0.263226

ATOM C13 lca 0.219139

ATOM C14 lca 0.056464

ATOM C15 lc3 -0.224743

ATOM H1 lhn 0.396704

ATOM H2 lh4 0.215196

ATOM H3 lhc 0.070140

ATOM H4 lhc 0.070140

ATOM H5 lhx 0.159003

ATOM H6 lhn 0.303609

ATOM H7 lhx 0.286079

ATOM H8 lhx 0.286079

ATOM H9 lhc 0.104687

ATOM H10 lha 0.170235

ATOM H11 lha 0.165798

ATOM H12 lha 0.162944

ATOM H13 lha 0.187989

ATOM H14 lhx 0.141964

ATOM H15 lhx 0.141964

ATOM H16 lhx 0.141964

GROUP

ATOM C16 lc 0.682278

ATOM O1 lo -0.580329

ATOM N3 ln -0.408838

ATOM C17 lc3 0.099247

ATOM C18 lc3 -0.172798

ATOM C19 lc3 0.099247

ATOM C20 lc3 -0.172798

ATOM H17 lh1 0.059829

ATOM H18 lh1 0.059829

ATOM H19 lhc 0.058280

ATOM H20 lhc 0.058280

ATOM H21 lhc 0.058280

ATOM H22 lh1 0.059829

ATOM H23 lh1 0.059829

ATOM H24 lhc 0.058280

ATOM H25 lhc 0.058280

ATOM H26 lhc 0.058280

BOND N1 C1 ! dist 1.3753

BOND N1 C13 ! dist 1.3730

BOND N1 H1 ! dist 0.9936

BOND C1 C2 ! dist 1.3513

BOND C1 H2 ! dist 1.0710

BOND C2 C3 ! dist 1.4983

BOND C2 C14 ! dist 1.4210

BOND C3 C4 ! dist 1.5521

BOND C3 H3 ! dist 1.0897

BOND C3 H4 ! dist 1.0830

BOND C4 N2 ! dist 1.5175

BOND C4 C8 ! dist 1.5333

BOND C4 H5 ! dist 1.0833

BOND N2 C5 ! dist 1.5020

BOND N2 C15 ! dist 1.4939

BOND N2 H6 ! dist 1.0088

BOND C5 C6 ! dist 1.5188

BOND C5 H7 ! dist 1.0796

BOND C5 H8 ! dist 1.0769

BOND C6 C7 ! dist 1.5100

BOND C6 C16 ! dist 1.5411

BOND C6 H9 ! dist 1.0863

BOND C7 C8 ! dist 1.3242

BOND C7 H10 ! dist 1.0739

BOND C8 C9 ! dist 1.4847

BOND C9 C10 ! dist 1.3792

BOND C9 C14 ! dist 1.3896

BOND C10 C11 ! dist 1.4061

BOND C10 H11 ! dist 1.0752

BOND C11 C12 ! dist 1.3806

BOND C11 H12 ! dist 1.0745

BOND C12 C13 ! dist 1.3934

BOND C12 H13 ! dist 1.0749

BOND C13 C14 ! dist 1.3875

BOND C15 H14 ! dist 1.0760

BOND C15 H15 ! dist 1.0800

BOND C15 H16 ! dist 1.0789

BOND C16 O1 ! dist 1.2052

BOND C16 N3 ! dist 1.3418

BOND N3 C17 ! dist 1.4613

BOND N3 C19 ! dist 1.4701

BOND C17 C18 ! dist 1.5269

BOND C17 H17 ! dist 1.0822

BOND C17 H18 ! dist 1.0808

BOND C18 H19 ! dist 1.0854

BOND C18 H20 ! dist 1.0858

BOND C18 H21 ! dist 1.0833

BOND C19 C20 ! dist 1.5240

BOND C19 H22 ! dist 1.0817

BOND C19 H23 ! dist 1.0782

BOND C20 H24 ! dist 1.0850

BOND C20 H25 ! dist 1.0828

BOND C20 H26 ! dist 1.0855

ANGL N1 C1 C2 ! angle 109.4432

ANGL N1 C1 H2 ! angle 120.0153

ANGL N1 C13 C12 ! angle 133.9955

ANGL N1 C13 C14 ! angle 106.0941

ANGL C1 N1 C13 ! angle 109.4986

ANGL C1 N1 H1 ! angle 124.5972

ANGL C1 C2 C3 ! angle 135.1931

ANGL C1 C2 C14 ! angle 106.1710

ANGL C2 C1 H2 ! angle 130.5403

ANGL C2 C3 C4 ! angle 108.5341

ANGL C2 C3 H3 ! angle 110.6293

ANGL C2 C3 H4 ! angle 111.0958

ANGL C2 C14 C9 ! angle 128.0029

ANGL C2 C14 C13 ! angle 108.7911

ANGL C3 C2 C14 ! angle 118.6293

ANGL C3 C4 N2 ! angle 109.0449

ANGL C3 C4 C8 ! angle 113.6130

ANGL C3 C4 H5 ! angle 109.1609

ANGL C4 C3 H3 ! angle 108.5930

ANGL C4 C3 H4 ! angle 110.3968

ANGL C4 N2 C5 ! angle 112.6363

ANGL C4 N2 C15 ! angle 112.8902

ANGL C4 N2 H6 ! angle 106.7054

ANGL C4 C8 C7 ! angle 123.0242

ANGL C4 C8 C9 ! angle 113.5819

ANGL N2 C4 C8 ! angle 110.5523

ANGL N2 C4 H5 ! angle 105.3800

ANGL N2 C5 C6 ! angle 110.0178

ANGL N2 C5 H7 ! angle 107.4750

ANGL N2 C5 H8 ! angle 107.6363

ANGL N2 C15 H14 ! angle 110.2668

ANGL N2 C15 H15 ! angle 108.3661

ANGL N2 C15 H16 ! angle 108.5954

ANGL C5 N2 C15 ! angle 109.5614

ANGL C5 N2 H6 ! angle 107.4882

ANGL C5 C6 C7 ! angle 109.3831

ANGL C5 C6 C16 ! angle 107.3402

ANGL C5 C6 H9 ! angle 109.4278

ANGL C6 C5 H7 ! angle 111.8197

ANGL C6 C5 H8 ! angle 110.7998

ANGL C6 C7 C8 ! angle 124.2526

ANGL C6 C7 H10 ! angle 117.0804

ANGL C6 C16 O1 ! angle 117.7455

ANGL C6 C16 N3 ! angle 118.8528

ANGL C7 C6 C16 ! angle 110.4020

ANGL C7 C6 H9 ! angle 110.3583

ANGL C7 C8 C9 ! angle 123.3911

ANGL C8 C4 H5 ! angle 108.7552

ANGL C8 C7 H10 ! angle 118.6367

ANGL C8 C9 C10 ! angle 127.4960

ANGL C8 C9 C14 ! angle 115.4560

ANGL C9 C10 C11 ! angle 119.9982

ANGL C9 C10 H11 ! angle 121.2385

ANGL C9 C14 C13 ! angle 123.1983

ANGL C10 C9 C14 ! angle 117.0255

ANGL C10 C11 C12 ! angle 122.7864

ANGL C10 C11 H12 ! angle 118.4169

ANGL C11 C10 H11 ! angle 118.7463

ANGL C11 C12 C13 ! angle 117.0730

ANGL C11 C12 H13 ! angle 120.7039

ANGL C12 C11 H12 ! angle 118.7901

ANGL C12 C13 C14 ! angle 119.9093

ANGL C13 N1 H1 ! angle 125.9013

ANGL C13 C12 H13 ! angle 122.2203

ANGL C15 N2 H6 ! angle 107.2322

ANGL C16 C6 H9 ! angle 109.8717

ANGL C16 N3 C17 ! angle 125.7047

ANGL C16 N3 C19 ! angle 117.4011

ANGL O1 C16 N3 ! angle 123.3971

ANGL N3 C17 C18 ! angle 113.3349

ANGL N3 C17 H17 ! angle 107.1056

ANGL N3 C17 H18 ! angle 110.2610

ANGL N3 C19 C20 ! angle 112.6052

ANGL N3 C19 H22 ! angle 107.1764

ANGL N3 C19 H23 ! angle 108.3888

ANGL C17 N3 C19 ! angle 116.8850

ANGL C17 C18 H19 ! angle 110.1494

ANGL C17 C18 H20 ! angle 111.4038

ANGL C17 C18 H21 ! angle 111.3140

ANGL C18 C17 H17 ! angle 109.8598

ANGL C18 C17 H18 ! angle 110.2334

ANGL C19 C20 H24 ! angle 109.9804

ANGL C19 C20 H25 ! angle 110.6404

ANGL C19 C20 H26 ! angle 111.4165

ANGL C20 C19 H22 ! angle 110.4435

ANGL C20 C19 H23 ! angle 110.3281

ANGL H3 C3 H4 ! angle 107.5647

ANGL H7 C5 H8 ! angle 108.9455

ANGL H14 C15 H15 ! angle 110.2648

ANGL H14 C15 H16 ! angle 109.4673

ANGL H15 C15 H16 ! angle 109.8519

ANGL H17 C17 H18 ! angle 105.7216

ANGL H19 C18 H20 ! angle 108.1356

ANGL H19 C18 H21 ! angle 107.9830

ANGL H20 C18 H21 ! angle 107.7214

ANGL H22 C19 H23 ! angle 107.7224

ANGL H24 C20 H25 ! angle 108.1470

ANGL H24 C20 H26 ! angle 107.8451

ANGL H25 C20 H26 ! angle 108.7082

DIHE C13 N1 C1 C2 ! dihe -0.4715

DIHE H1 N1 C1 C2 ! dihe -179.8779

DIHE C13 N1 C1 H2 ! dihe 179.8900

DIHE H1 N1 C1 H2 ! dihe 0.4836

DIHE C1 N1 C13 C12 ! dihe -179.3066

DIHE H1 N1 C13 C12 ! dihe 0.0902

DIHE C1 N1 C13 C14 ! dihe 0.3048

DIHE H1 N1 C13 C14 ! dihe 179.7015

DIHE N1 C1 C2 C3 ! dihe -178.5838

DIHE H2 C1 C2 C3 ! dihe 1.0043

DIHE N1 C1 C2 C14 ! dihe 0.4284

DIHE H2 C1 C2 C14 ! dihe -179.9835

DIHE C1 C2 C3 C4 ! dihe -153.8976

DIHE C14 C2 C3 C4 ! dihe 27.1833

DIHE C1 C2 C3 H3 ! dihe 87.0548

DIHE C14 C2 C3 H3 ! dihe -91.8644

DIHE C1 C2 C3 H4 ! dihe -32.3420

DIHE C14 C2 C3 H4 ! dihe 148.7389

DIHE C1 C2 C14 C9 ! dihe 178.7625

DIHE C3 C2 C14 C9 ! dihe -2.0306

DIHE C1 C2 C14 C13 ! dihe -0.2399

DIHE C3 C2 C14 C13 ! dihe 178.9671

DIHE C2 C3 C4 N2 ! dihe -175.0091

DIHE H3 C3 C4 N2 ! dihe -54.6886

DIHE H4 C3 C4 N2 ! dihe 63.0077

DIHE C2 C3 C4 C8 ! dihe -51.2131

DIHE H3 C3 C4 C8 ! dihe 69.1075

DIHE H4 C3 C4 C8 ! dihe -173.1963

DIHE C2 C3 C4 H5 ! dihe 70.3467

DIHE H3 C3 C4 H5 ! dihe -169.3328

DIHE H4 C3 C4 H5 ! dihe -51.6365

DIHE C3 C4 N2 C5 ! dihe 165.7671

DIHE C8 C4 N2 C5 ! dihe 40.1770

DIHE H5 C4 N2 C5 ! dihe -77.1627

DIHE C3 C4 N2 C15 ! dihe -69.4941

DIHE C8 C4 N2 C15 ! dihe 164.9157

DIHE H5 C4 N2 C15 ! dihe 47.5760

DIHE C3 C4 N2 H6 ! dihe 48.0513

DIHE C8 C4 N2 H6 ! dihe -77.5389

DIHE H5 C4 N2 H6 ! dihe 165.1214

DIHE C3 C4 C8 C7 ! dihe -129.1667

DIHE N2 C4 C8 C7 ! dihe -6.1942

DIHE H5 C4 C8 C7 ! dihe 109.0476

DIHE C3 C4 C8 C9 ! dihe 51.4208

DIHE N2 C4 C8 C9 ! dihe 174.3934

DIHE H5 C4 C8 C9 ! dihe -70.3648

DIHE C4 N2 C5 C6 ! dihe -64.5349

DIHE C15 N2 C5 C6 ! dihe 168.9228

DIHE H6 N2 C5 C6 ! dihe 52.7181

DIHE C4 N2 C5 H7 ! dihe 173.4987

DIHE C15 N2 C5 H7 ! dihe 46.9565

DIHE H6 N2 C5 H7 ! dihe -69.2482

DIHE C4 N2 C5 H8 ! dihe 56.2888

DIHE C15 N2 C5 H8 ! dihe -70.2534

DIHE H6 N2 C5 H8 ! dihe 173.5419

DIHE C4 N2 C15 H14 ! dihe 57.8961

DIHE C5 N2 C15 H14 ! dihe -175.7058

DIHE H6 N2 C15 H14 ! dihe -59.3391

DIHE C4 N2 C15 H15 ! dihe -62.8761

DIHE C5 N2 C15 H15 ! dihe 63.5220

DIHE H6 N2 C15 H15 ! dihe 179.8887

DIHE C4 N2 C15 H16 ! dihe 177.8343

DIHE C5 N2 C15 H16 ! dihe -55.7676

DIHE H6 N2 C15 H16 ! dihe 60.5991

DIHE N2 C5 C6 C7 ! dihe 50.5236

DIHE H7 C5 C6 C7 ! dihe 169.8724

DIHE H8 C5 C6 C7 ! dihe -68.3793

DIHE N2 C5 C6 C16 ! dihe 170.3251

DIHE H7 C5 C6 C16 ! dihe -70.3260

DIHE H8 C5 C6 C16 ! dihe 51.4223

DIHE N2 C5 C6 H9 ! dihe -70.4838

DIHE H7 C5 C6 H9 ! dihe 48.8650

DIHE H8 C5 C6 H9 ! dihe 170.6134

DIHE C5 C6 C7 C8 ! dihe -17.8300

DIHE C16 C6 C7 C8 ! dihe -135.7317

DIHE H9 C6 C7 C8 ! dihe 102.6107

DIHE C5 C6 C7 H10 ! dihe 160.1342

DIHE C16 C6 C7 H10 ! dihe 42.2325

DIHE H9 C6 C7 H10 ! dihe -79.4250

DIHE C5 C6 C16 O1 ! dihe -23.8245

DIHE C7 C6 C16 O1 ! dihe 95.3248

DIHE H9 C6 C16 O1 ! dihe -142.7302

DIHE C5 C6 C16 N3 ! dihe 156.9304

DIHE C7 C6 C16 N3 ! dihe -83.9203

DIHE H9 C6 C16 N3 ! dihe 38.0247

DIHE C6 C7 C8 C4 ! dihe -4.7193

DIHE H10 C7 C8 C4 ! dihe 177.3459

DIHE C6 C7 C8 C9 ! dihe 174.6357

DIHE H10 C7 C8 C9 ! dihe -3.2990

DIHE C4 C8 C9 C10 ! dihe 153.9871

DIHE C7 C8 C9 C10 ! dihe -25.4229

DIHE C4 C8 C9 C14 ! dihe -24.2233

DIHE C7 C8 C9 C14 ! dihe 156.3667

DIHE C8 C9 C10 C11 ! dihe -179.1234

DIHE C14 C9 C10 C11 ! dihe -0.9373

DIHE C8 C9 C10 H11 ! dihe -0.6468

DIHE C14 C9 C10 H11 ! dihe 177.5393

DIHE C8 C9 C14 C2 ! dihe -0.2611

DIHE C10 C9 C14 C2 ! dihe -178.6673

DIHE C8 C9 C14 C13 ! dihe 178.6102

DIHE C10 C9 C14 C13 ! dihe 0.2040

DIHE C9 C10 C11 C12 ! dihe 0.9516

DIHE H11 C10 C11 C12 ! dihe -177.5628

DIHE C9 C10 C11 H12 ! dihe -179.9927

DIHE H11 C10 C11 H12 ! dihe 1.4929

DIHE C10 C11 C12 C13 ! dihe -0.1604

DIHE H12 C11 C12 C13 ! dihe -179.2127

DIHE C10 C11 C12 H13 ! dihe 179.2527

DIHE H12 C11 C12 H13 ! dihe 0.2004

DIHE C11 C12 C13 N1 ! dihe 178.9870

DIHE H13 C12 C13 N1 ! dihe -0.4165

DIHE C11 C12 C13 C14 ! dihe -0.5822

DIHE H13 C12 C13 C14 ! dihe -179.9856

DIHE N1 C13 C14 C2 ! dihe -0.0395

DIHE C12 C13 C14 C2 ! dihe 179.6379

DIHE N1 C13 C14 C9 ! dihe -179.1001

DIHE C12 C13 C14 C9 ! dihe 0.5774

DIHE C6 C16 N3 C17 ! dihe -2.9465

DIHE O1 C16 N3 C17 ! dihe 177.8537

DIHE C6 C16 N3 C19 ! dihe 178.1916

DIHE O1 C16 N3 C19 ! dihe -1.0082

DIHE C16 N3 C17 C18 ! dihe -94.0723

DIHE C19 N3 C17 C18 ! dihe 84.7949

DIHE C16 N3 C17 H17 ! dihe 144.6045

DIHE C19 N3 C17 H17 ! dihe -36.5284

DIHE C16 N3 C17 H18 ! dihe 30.0345

DIHE C19 N3 C17 H18 ! dihe -151.0983

DIHE C16 N3 C19 C20 ! dihe -85.6776

DIHE C17 N3 C19 C20 ! dihe 95.3585

DIHE C16 N3 C19 H22 ! dihe 152.6736

DIHE C17 N3 C19 H22 ! dihe -26.2902

DIHE C16 N3 C19 H23 ! dihe 36.6643

DIHE C17 N3 C19 H23 ! dihe -142.2996

DIHE N3 C17 C18 H19 ! dihe -177.5392

DIHE H17 C17 C18 H19 ! dihe -57.7765

DIHE H18 C17 C18 H19 ! dihe 58.3389

DIHE N3 C17 C18 H20 ! dihe 62.4644

DIHE H17 C17 C18 H20 ! dihe -177.7728

DIHE H18 C17 C18 H20 ! dihe -61.6575

DIHE N3 C17 C18 H21 ! dihe -57.7923

DIHE H17 C17 C18 H21 ! dihe 61.9704

DIHE H18 C17 C18 H21 ! dihe 178.0858

DIHE N3 C19 C20 H24 ! dihe -178.0851

DIHE H22 C19 C20 H24 ! dihe -58.3100

DIHE H23 C19 C20 H24 ! dihe 60.6723

DIHE N3 C19 C20 H25 ! dihe 62.5026

DIHE H22 C19 C20 H25 ! dihe -177.7223

DIHE H23 C19 C20 H25 ! dihe -58.7400

DIHE N3 C19 C20 H26 ! dihe -58.5561

DIHE H22 C19 C20 H26 ! dihe 61.2189

DIHE H23 C19 C20 H26 ! dihe -179.7987

IMPH C13 C1 N1 H1

IMPH C2 H2 C1 N1

IMPH C3 C14 C2 C1

IMPH C6 C8 C7 H10

IMPH C7 C4 C8 C9

IMPH C10 C14 C9 C8

IMPH C9 C11 C10 H11

IMPH C10 C12 C11 H12

IMPH C11 C13 C12 H13

IMPH C12 C14 C13 N1

IMPH C9 C13 C14 C2

IMPH C6 N3 C16 O1

IMPH C16 C17 N3 C19

***Parameter file for LSD***

* Forlce Field Parameter File.

*

BOND

lcc lna 438.80 1.371

lca lna 470.30 1.350

lhn lna 406.60 1.011

lcc lcd 504.00 1.371

lcc lh4 350.10 1.083

lc3 lcd 337.30 1.499

lca lcd 411.70 1.434

lc3 lc3 303.10 1.535

lc3 lhc 337.30 1.092

lc3 ln4 293.60 1.499

lc3 lce 331.30 1.505

lc3 lhx 338.70 1.091

lhn ln4 369.00 1.033

lc2 lc3 328.30 1.508

lc lc3 328.30 1.508

lc2 lce 560.50 1.339

lc2 lha 344.30 1.087

lca lce 366.00 1.472

lca lca 478.40 1.387

lca lha 344.30 1.087

lc lo 648.00 1.214

lc ln 478.20 1.345

lc3 ln 330.60 1.460

lc3 lh1 335.90 1.093

ANGLE

lna lcc lcd 72.900 109.420

lna lcc lh4 50.200 119.660

lna lca lca 70.200 118.340

lcc lna lca 68.500 113.150

lcc lna lhn 47.200 124.660

lcc lcd lc3 64.800 119.450

lcc lcd lca 68.200 113.510

lcd lcc lh4 47.200 129.110

lcd lc3 lc3 64.700 108.100

lcd lc3 lhc 47.200 110.860

lcd lca lca 66.000 120.100

lc3 lcd lca 64.100 117.810

lc3 lc3 ln4 66.000 108.930

lc3 lc3 lce 63.700 110.960 ! same as lc2 lc3 lc3

lc3 lc3 lhx 46.000 111.740

lc3 lc3 lhc 46.400 110.050

lc3 ln4 lc3 62.800 110.640

lc3 ln4 lhn 46.200 110.110

lc3 lce lc2 64.300 122.890

lc3 lce lca 63.200 117.620

ln4 lc3 lce 65.845 111.515 ! Calculated with empirilcal approach

ln4 lc3 lhx 49.000 107.910

lc3 lc3 lc2 63.700 110.960

lc3 lc3 lc 63.800 110.530

lc3 lc2 lce 65.700 117.400

lc3 lc2 lha 45.700 117.300

lc3 lc lo 68.000 123.110

lc3 lc ln 67.900 115.150

lc2 lc3 lc 64.600 109.730

lc2 lc3 lhc 47.000 110.490

lc2 lce lca 65.200 123.080

lce lc3 lhx 47.000 110.980 ! same as lce lc3 lhc

lce lc2 lha 49.600 121.190

lce lca lca 64.900 120.660

lca lca lca 67.200 119.970

lca lca lha 48.500 120.010

lca lna lhn 48.200 122.770

lc lc3 lhc 47.200 109.680

lc ln lc3 63.900 121.350

lo lc ln 75.800 122.030

ln lc3 lc3 65.900 112.130

ln lc3 lh1 49.800 109.320

lc3 ln lc3 64.000 112.620

lc3 lc3 lh1 46.400 110.070

lhc lc3 lhc 39.400 108.350

lhx lc3 lhx 39.000 110.740

lh1 lc3 lh1 39.200 109.550

DIHEDRAL

X lcc lna X 1.700 2 180.0

X lca lna X 0.300 2 180.0

X lcc lcd X 4.000 2 180.0

X lc3 lcd X 0.000 3 0.0

lcc lcd lca lca 2.550 2 180.0 ! same as X lc2 lca X

lc3 lcd lca lca 2.550 2 180.0 ! same as X lc2 lca X

X lc3 lc3 X 0.156 3 0.0

X lc3 ln4 X 0.156 3 0.0

lc3 lc3 lce lc2 0.000 2 0.0 ! same as X lc2 lc3 X

ln4 lc3 lce lc2 0.000 2 0.0 ! same as X lc2 lc3 X

lhx lc3 lce lc2 0.000 2 0.0 ! same as X lc2 lc3 X

lc3 lc3 lce lca 0.000 2 0.0 ! same as X lc2 lc3 X

ln4 lc3 lce lca 0.000 2 0.0 ! same as X lc2 lc3 X

lhx lc3 lce lca 0.000 2 0.0 ! same as X lc2 lc3 X

X lc2 lc3 X 0.000 2 0.0

X lc lc3 X 0.000 2 180.0

lhc lc3 lc lo 0.800 1 0.0

lhc lc3 lc lo 0.080 3 180.0

lc3 lc3 lc ln 0.100 4 0.0

lc3 lc3 lc ln 0.070 2 0.0

X lc2 lce X 6.650 2 180.0

lc3 lce lca lca 2.550 2 180.0 ! same as X lc2 lca X

lc2 lce lca lca 2.550 2 180.0 ! same as X lc2 lca X

X lca lca X 3.625 2 180.0

X lc ln X 2.500 2 180.0

X lc3 ln X 0.000 2 0.0

lc3 lc3 ln lc 0.500 4 180.0

lc3 lc3 ln lc 0.150 3 180.0

lc3 lc3 ln lc 0.530 1 0.0

IMPHI

X X lna lhn 1.100 0 180.0

lcd lh4 lcc lna 1.100 0 180.0 ! Using default value

lc3 lca lcd lcc 1.100 0 180.0 ! Using default value

lc3 lce lc2 lha 1.100 0 180.0 ! Using default value

lc2 lc3 lce lca 1.100 0 180.0 ! Using default value

lca lca lca lce 1.100 0 180.0 ! Using default value

X X lca lha 1.100 0 180.0

lca lca lca lna 1.100 0 180.0 ! Using default value

lca lca lca lcd 1.100 0 180.0 ! Using default value

X X lc lo 10.500 0 180.0

X lc3 ln lc3 1.100 0 180.0

NONBONDED NBXMOD 5 GROUP SWITCH CDIEL -

CUTNB 14.0 CTOFNB 12.0 CTONNB 10.0 EPS 1.0 E14FAC 0.83333333 WMIN 1.4

! Emin Rmin/2 Emin/2 Rmin (for 1-4's)

! (klcal/mol) (A)

lna 0.00 -0.1700 1.8240 0.00 -0.0850 1.8240

lcc 0.00 -0.0860 1.9080 0.00 -0.0430 1.9080

lcd 0.00 -0.0860 1.9080 0.00 -0.0430 1.9080

lc3 0.00 -0.1094 1.9080 0.00 -0.0547 1.9080

ln4 0.00 -0.1700 1.8240 0.00 -0.0850 1.8240

lc2 0.00 -0.0860 1.9080 0.00 -0.0430 1.9080

lce 0.00 -0.0860 1.9080 0.00 -0.0430 1.9080

lca 0.00 -0.0860 1.9080 0.00 -0.0430 1.9080

lc 0.00 -0.0860 1.9080 0.00 -0.0430 1.9080

lo 0.00 -0.2100 1.6612 0.00 -0.1050 1.6612

ln 0.00 -0.1700 1.8240 0.00 -0.0850 1.8240

lhn 0.00 -0.0157 0.6000 0.00 -0.0078 0.6000

lh4 0.00 -0.0150 1.4090 0.00 -0.0075 1.4090

lhc 0.00 -0.0157 1.4870 0.00 -0.0078 1.4870

lhx 0.00 -0.0157 1.1000 0.00 -0.0078 1.1000

lha 0.00 -0.0150 1.4590 0.00 -0.0075 1.4590

lh1 0.00 -0.0157 1.3870 0.00 -0.0078 1.3870

***Topology file for KET***

* Topology File.

*

99 1

MASS 301 lca 12.010000

MASS 302 ln 14.010000

MASS 303 lc 12.010000

MASS 304 lo 16.000000

MASS 305 lc3 12.010000

MASS 306 ln4 14.010000

MASS 307 lf 19.000000

MASS 308 lha 1.008000

MASS 309 lhn 1.008000

MASS 310 lh1 1.008000

MASS 311 lhx 1.008000

MASS 312 lhc 1.008000

RESI Ket 1.000

GROUP

ATOM C1 lca -0.296815

ATOM C2 lca -0.009884

ATOM H1 lha 0.153986

ATOM C3 lca -0.234728

ATOM H2 lha 0.171015

ATOM C4 lca -0.003149

ATOM H3 lha 0.158239

ATOM C5 lca -0.350382

ATOM H4 lha 0.195082

ATOM C6 lca 0.425274

ATOM N1 ln -0.699757

ATOM H5 lhn 0.417204

ATOM C7 lc 0.749952

ATOM O2 lo -0.597216

ATOM N2 ln -0.158072

ATOM C8 lc 0.643682

ATOM O1 lo -0.549078

GROUP

ATOM C9 lc3 -0.155669

ATOM H6 lh1 0.134469

ATOM H7 lh1 0.134469

GROUP

ATOM C10 lc3 -0.419263

ATOM H8 lhx 0.210798

ATOM H9 lhx 0.210798

GROUP

ATOM N3 ln4 0.008466

ATOM H23 lhn 0.351961

ATOM C11 lc3 -0.170616

ATOM H10 lhx 0.148541

ATOM H11 lhx 0.148541

ATOM C12 lc3 0.116796

ATOM H12 lhc 0.019894

ATOM H13 lhc 0.019894

ATOM C13 lc3 -0.243043

ATOM H14 lhc 0.035438

ATOM C14 lc3 0.116796

ATOM H15 lhc 0.019894

ATOM H16 lhc 0.019894

ATOM C15 lc3 -0.170616

ATOM H17 lhx 0.148541

ATOM H18 lhx 0.148541

GROUP

ATOM C16 lc 0.633248

ATOM O3 lo -0.507103

GROUP

ATOM C17 lca -0.218661

ATOM C18 lca -0.031968

ATOM H19 lha 0.164206

ATOM C19 lca -0.366092

ATOM H20 lha 0.203795

ATOM C20 lca 0.515454

ATOM F1 lf -0.212696

ATOM C21 lca -0.366092

ATOM H21 lha 0.203795

ATOM C22 lca -0.031968

ATOM H22 lha 0.164206

BOND C1 C2 ! dist 1.3957

BOND C1 C6 ! dist 1.3885

BOND C1 C8 ! dist 1.4652

BOND C2 C3 ! dist 1.3748

BOND C2 H1 ! dist 1.0729

BOND C3 C4 ! dist 1.3944

BOND C3 H2 ! dist 1.0736

BOND C4 C5 ! dist 1.3792

BOND C4 H3 ! dist 1.0753

BOND C5 C6 ! dist 1.3896

BOND C5 H4 ! dist 1.0749

BOND C6 N1 ! dist 1.3899

BOND N1 C7 ! dist 1.3521

BOND N1 H5 ! dist 0.9974

BOND C7 N2 ! dist 1.3872

BOND C7 O2 ! dist 1.1997

BOND N2 C8 ! dist 1.3980

BOND N2 C9 ! dist 1.4503

BOND C8 O1 ! dist 1.1975

BOND C9 C10 ! dist 1.5290

BOND C9 H6 ! dist 1.0802

BOND C9 H7 ! dist 1.0762

BOND C10 N3 ! dist 1.5008

BOND C10 H8 ! dist 1.0806

BOND C10 H9 ! dist 1.0781

BOND N3 C11 ! dist 1.5058

BOND N3 C15 ! dist 1.5035

BOND N3 H23 ! dist 1.0104

BOND C11 C12 ! dist 1.5223

BOND C11 H10 ! dist 1.0821

BOND C11 H11 ! dist 1.0809

BOND C12 C13 ! dist 1.5315

BOND C12 H12 ! dist 1.0839

BOND C12 H13 ! dist 1.0839

BOND C13 C14 ! dist 1.5388

BOND C13 C16 ! dist 1.5339

BOND C13 H14 ! dist 1.0841

BOND C14 C15 ! dist 1.5240

BOND C14 H15 ! dist 1.0831

BOND C14 H16 ! dist 1.0862

BOND C15 H17 ! dist 1.0822

BOND C15 H18 ! dist 1.0796

BOND C16 O3 ! dist 1.1965

BOND C16 C17 ! dist 1.4878

BOND C17 C18 ! dist 1.3979

BOND C17 C22 ! dist 1.3932

BOND C18 C19 ! dist 1.3772

BOND C18 H19 ! dist 1.0730

BOND C19 C20 ! dist 1.3821

BOND C19 H20 ! dist 1.0736

BOND C20 C21 ! dist 1.3797

BOND C20 F1 ! dist 1.3178

BOND C21 C22 ! dist 1.3827

BOND C21 H21 ! dist 1.0739

BOND C22 H22 ! dist 1.0732

ANGL C1 C2 C3 ! angle 120.0613

ANGL C1 C2 H1 ! angle 118.5739

ANGL C1 C6 C5 ! angle 120.2533

ANGL C1 C6 N1 ! angle 118.8086

ANGL C1 C8 N2 ! angle 115.3585

ANGL C1 C8 O1 ! angle 125.0444

ANGL C2 C1 C6 ! angle 119.9852

ANGL C2 C1 C8 ! angle 120.3846

ANGL C2 C3 C4 ! angle 119.3514

ANGL C2 C3 H2 ! angle 120.5079

ANGL C3 C2 H1 ! angle 121.3643

ANGL C3 C4 C5 ! angle 121.3653

ANGL C3 C4 H3 ! angle 119.6287

ANGL C4 C3 H2 ! angle 120.1407

ANGL C4 C5 C6 ! angle 118.9831

ANGL C4 C5 H4 ! angle 120.7117

ANGL C5 C4 H3 ! angle 119.0059

ANGL C5 C6 N1 ! angle 120.9344

ANGL C6 C1 C8 ! angle 119.6303

ANGL C6 C5 H4 ! angle 120.3052

ANGL C6 N1 C7 ! angle 124.9950

ANGL C6 N1 H5 ! angle 120.0449

ANGL N1 C7 N2 ! angle 115.6285

ANGL N1 C7 O2 ! angle 123.3309

ANGL C7 N1 H5 ! angle 114.9443

ANGL C7 N2 C8 ! angle 125.2856

ANGL C7 N2 C9 ! angle 116.6218

ANGL N2 C7 O2 ! angle 121.0385

ANGL N2 C8 O1 ! angle 119.5971

ANGL N2 C9 C10 ! angle 109.7366

ANGL N2 C9 H6 ! angle 107.9502

ANGL N2 C9 H7 ! angle 107.2390

ANGL C8 N2 C9 ! angle 118.0803

ANGL C9 C10 N3 ! angle 112.5573

ANGL C9 C10 H8 ! angle 111.2117

ANGL C9 C10 H9 ! angle 109.3092

ANGL C10 C9 H6 ! angle 110.4212

ANGL C10 C9 H7 ! angle 111.6494

ANGL C10 N3 C11 ! angle 111.1693

ANGL C10 N3 C15 ! angle 113.3797

ANGL C10 N3 H23 ! angle 106.9220

ANGL N3 C10 H8 ! angle 107.4877

ANGL N3 C10 H9 ! angle 106.4381

ANGL N3 C11 C12 ! angle 111.0396

ANGL N3 C11 H10 ! angle 106.2272

ANGL N3 C11 H11 ! angle 107.0161

ANGL N3 C15 C14 ! angle 111.1625

ANGL N3 C15 H17 ! angle 106.2238

ANGL N3 C15 H18 ! angle 107.9048

ANGL C11 N3 C15 ! angle 110.7736

ANGL C11 N3 H23 ! angle 106.6925

ANGL C11 C12 C13 ! angle 111.8172

ANGL C11 C12 H12 ! angle 107.7454

ANGL C11 C12 H13 ! angle 110.2168

ANGL C12 C11 H10 ! angle 112.2942

ANGL C12 C11 H11 ! angle 111.3429

ANGL C12 C13 C14 ! angle 109.3965

ANGL C12 C13 C16 ! angle 110.2166

ANGL C12 C13 H14 ! angle 108.6815

ANGL C13 C12 H12 ! angle 110.6653

ANGL C13 C12 H13 ! angle 109.7860

ANGL C13 C14 C15 ! angle 112.1187

ANGL C13 C14 H15 ! angle 111.2552

ANGL C13 C14 H16 ! angle 109.3139

ANGL C13 C16 O3 ! angle 117.9338

ANGL C13 C16 C17 ! angle 120.6142

ANGL C14 C13 C16 ! angle 108.9928

ANGL C14 C13 H14 ! angle 108.9968

ANGL C14 C15 H17 ! angle 111.9980

ANGL C14 C15 H18 ! angle 110.8565

ANGL C15 N3 H23 ! angle 107.5344

ANGL C15 C14 H15 ! angle 107.6065

ANGL C15 C14 H16 ! angle 109.6451

ANGL C16 C13 H14 ! angle 110.5340

ANGL C16 C17 C18 ! angle 117.4262

ANGL C16 C17 C22 ! angle 123.8086

ANGL O3 C16 C17 ! angle 121.4122

ANGL C17 C18 C19 ! angle 120.9951

ANGL C17 C18 H19 ! angle 118.8562

ANGL C17 C22 C21 ! angle 120.9420

ANGL C17 C22 H22 ! angle 121.2964

ANGL C18 C17 C22 ! angle 118.7641

ANGL C18 C19 C20 ! angle 118.5252

ANGL C18 C19 H20 ! angle 121.8209

ANGL C19 C18 H19 ! angle 120.1486

ANGL C19 C20 C21 ! angle 122.2866

ANGL C19 C20 F1 ! angle 118.9468

ANGL C20 C19 H20 ! angle 119.6539

ANGL C20 C21 C22 ! angle 118.4867

ANGL C20 C21 H21 ! angle 119.6968

ANGL C21 C20 F1 ! angle 118.7665

ANGL C21 C22 H22 ! angle 117.7612

ANGL C22 C21 H21 ! angle 121.8160

ANGL H6 C9 H7 ! angle 109.7241

ANGL H8 C10 H9 ! angle 109.6969

ANGL H10 C11 H11 ! angle 108.6482

ANGL H12 C12 H13 ! angle 106.4539

ANGL H15 C14 H16 ! angle 106.7441

ANGL H17 C15 H18 ! angle 108.4901

DIHE C6 C1 C2 C3 ! dihe -0.2091

DIHE C8 C1 C2 C3 ! dihe 179.7343

DIHE C6 C1 C2 H1 ! dihe -179.9608

DIHE C8 C1 C2 H1 ! dihe -0.0173

DIHE C2 C1 C6 C5 ! dihe 0.0295

DIHE C8 C1 C6 C5 ! dihe -179.9144

DIHE C2 C1 C6 N1 ! dihe -179.2775

DIHE C8 C1 C6 N1 ! dihe 0.7786

DIHE C2 C1 C8 N2 ! dihe -177.0884

DIHE C6 C1 C8 N2 ! dihe 2.8553

DIHE C2 C1 C8 O1 ! dihe 2.9628

DIHE C6 C1 C8 O1 ! dihe -177.0936

DIHE C1 C2 C3 C4 ! dihe 0.2212

DIHE H1 C2 C3 C4 ! dihe 179.9657

DIHE C1 C2 C3 H2 ! dihe -179.7969

DIHE H1 C2 C3 H2 ! dihe -0.0523

DIHE C2 C3 C4 C5 ! dihe -0.0564

DIHE H2 C3 C4 C5 ! dihe 179.9615

DIHE C2 C3 C4 H3 ! dihe -179.9721

DIHE H2 C3 C4 H3 ! dihe 0.0458

DIHE C3 C4 C5 C6 ! dihe -0.1211

DIHE H3 C4 C5 C6 ! dihe 179.7952

DIHE C3 C4 C5 H4 ! dihe 179.9757

DIHE H3 C4 C5 H4 ! dihe -0.1081

DIHE C4 C5 C6 C1 ! dihe 0.1333

DIHE H4 C5 C6 C1 ! dihe -179.9631

DIHE C4 C5 C6 N1 ! dihe 179.4253

DIHE H4 C5 C6 N1 ! dihe -0.6710

DIHE C1 C6 N1 C7 ! dihe -1.4536

DIHE C5 C6 N1 C7 ! dihe 179.2443

DIHE C1 C6 N1 H5 ! dihe 177.0277

DIHE C5 C6 N1 H5 ! dihe -2.2743

DIHE C6 N1 C7 N2 ! dihe -1.6968

DIHE H5 N1 C7 N2 ! dihe 179.7531

DIHE C6 N1 C7 O2 ! dihe 177.7721

DIHE H5 N1 C7 O2 ! dihe -0.7781

DIHE N1 C7 N2 C8 ! dihe 5.9562

DIHE O2 C7 N2 C8 ! dihe -173.5259

DIHE N1 C7 N2 C9 ! dihe -175.3466

DIHE O2 C7 N2 C9 ! dihe 5.1713

DIHE C7 N2 C8 C1 ! dihe -6.4955

DIHE C9 N2 C8 C1 ! dihe 174.8246

DIHE C7 N2 C8 O1 ! dihe 173.4564

DIHE C9 N2 C8 O1 ! dihe -5.2235

DIHE C7 N2 C9 C10 ! dihe -84.8688

DIHE C8 N2 C9 C10 ! dihe 93.9258

DIHE C7 N2 C9 H6 ! dihe 35.5216

DIHE C8 N2 C9 H6 ! dihe -145.6837

DIHE C7 N2 C9 H7 ! dihe 153.6838

DIHE C8 N2 C9 H7 ! dihe -27.5215

DIHE N2 C9 C10 N3 ! dihe 167.6199

DIHE H6 C9 C10 N3 ! dihe 48.7409

DIHE H7 C9 C10 N3 ! dihe -73.6191

DIHE N2 C9 C10 H8 ! dihe -71.6993

DIHE H6 C9 C10 H8 ! dihe 169.4218

DIHE H7 C9 C10 H8 ! dihe 47.0618

DIHE N2 C9 C10 H9 ! dihe 49.5708

DIHE H6 C9 C10 H9 ! dihe -69.3081

DIHE H7 C9 C10 H9 ! dihe 168.3318

DIHE C9 C10 N3 C11 ! dihe -168.4774

DIHE H8 C10 N3 C11 ! dihe 68.7269

DIHE H9 C10 N3 C11 ! dihe -48.7501

DIHE C9 C10 N3 C15 ! dihe 65.9466

DIHE H8 C10 N3 C15 ! dihe -56.8491

DIHE H9 C10 N3 C15 ! dihe -174.3260

DIHE C9 C10 N3 H23 ! dihe -52.3875

DIHE H8 C10 N3 H23 ! dihe -175.1832

DIHE H9 C10 N3 H23 ! dihe 67.3399

DIHE C10 N3 C11 C12 ! dihe 175.4096

DIHE C15 N3 C11 C12 ! dihe -57.5751

DIHE H23 N3 C11 C12 ! dihe 59.1785

DIHE C10 N3 C11 H10 ! dihe -62.2278

DIHE C15 N3 C11 H10 ! dihe 64.7875

DIHE H23 N3 C11 H10 ! dihe -178.4589

DIHE C10 N3 C11 H11 ! dihe 53.7065

DIHE C15 N3 C11 H11 ! dihe -179.2782

DIHE H23 N3 C11 H11 ! dihe -62.5246

DIHE C10 N3 C15 C14 ! dihe -177.3110

DIHE C11 N3 C15 C14 ! dihe 56.9018

DIHE H23 N3 C15 C14 ! dihe -59.3317

DIHE C10 N3 C15 H17 ! dihe 60.6184

DIHE C11 N3 C15 H17 ! dihe -65.1688

DIHE H23 N3 C15 H17 ! dihe 178.5977

DIHE C10 N3 C15 H18 ! dihe -55.5566

DIHE C11 N3 C15 H18 ! dihe 178.6562

DIHE H23 N3 C15 H18 ! dihe 62.4227

DIHE N3 C11 C12 C13 ! dihe 57.0625

DIHE H10 C11 C12 C13 ! dihe -61.7085

DIHE H11 C11 C12 C13 ! dihe 176.2018

DIHE N3 C11 C12 H12 ! dihe 178.8813

DIHE H10 C11 C12 H12 ! dihe 60.1103

DIHE H11 C11 C12 H12 ! dihe -61.9794

DIHE N3 C11 C12 H13 ! dihe -65.3482

DIHE H10 C11 C12 H13 ! dihe 175.8807

DIHE H11 C11 C12 H13 ! dihe 53.7910

DIHE C11 C12 C13 C14 ! dihe -54.2999

DIHE H12 C12 C13 C14 ! dihe -174.4238

DIHE H13 C12 C13 C14 ! dihe 68.3566

DIHE C11 C12 C13 C16 ! dihe -174.1243

DIHE H12 C12 C13 C16 ! dihe 65.7519

DIHE H13 C12 C13 C16 ! dihe -51.4677

DIHE C11 C12 C13 H14 ! dihe 64.6041

DIHE H12 C12 C13 H14 ! dihe -55.5198

DIHE H13 C12 C13 H14 ! dihe -172.7393

DIHE C12 C13 C14 C15 ! dihe 53.7369

DIHE C16 C13 C14 C15 ! dihe 174.3102

DIHE H14 C13 C14 C15 ! dihe -64.9715

DIHE C12 C13 C14 H15 ! dihe 174.2967

DIHE C16 C13 C14 H15 ! dihe -65.1300

DIHE H14 C13 C14 H15 ! dihe 55.5883

DIHE C12 C13 C14 H16 ! dihe -68.0651

DIHE C16 C13 C14 H16 ! dihe 52.5083

DIHE H14 C13 C14 H16 ! dihe 173.2265

DIHE C12 C13 C16 O3 ! dihe 31.2247

DIHE C14 C13 C16 O3 ! dihe -88.8436

DIHE H14 C13 C16 O3 ! dihe 151.3856

DIHE C12 C13 C16 C17 ! dihe -151.0349

DIHE C14 C13 C16 C17 ! dihe 88.8968

DIHE H14 C13 C16 C17 ! dihe -30.8741

DIHE C13 C14 C15 N3 ! dihe -55.7707

DIHE H15 C14 C15 N3 ! dihe -178.4226

DIHE H16 C14 C15 N3 ! dihe 65.8417

DIHE C13 C14 C15 H17 ! dihe 62.8828

DIHE H15 C14 C15 H17 ! dihe -59.7692

DIHE H16 C14 C15 H17 ! dihe -175.5049

DIHE C13 C14 C15 H18 ! dihe -175.7889

DIHE H15 C14 C15 H18 ! dihe 61.5591

DIHE H16 C14 C15 H18 ! dihe -54.1766

DIHE C13 C16 C17 C18 ! dihe -177.5457

DIHE O3 C16 C17 C18 ! dihe 0.1151

DIHE C13 C16 C17 C22 ! dihe 2.0658

DIHE O3 C16 C17 C22 ! dihe 179.7265

DIHE C16 C17 C18 C19 ! dihe 179.7657

DIHE C22 C17 C18 C19 ! dihe 0.1340

DIHE C16 C17 C18 H19 ! dihe -0.3415

DIHE C22 C17 C18 H19 ! dihe -179.9733

DIHE C16 C17 C22 C21 ! dihe -179.6057

DIHE C18 C17 C22 C21 ! dihe 0.0009

DIHE C16 C17 C22 H22 ! dihe 0.6042

DIHE C18 C17 C22 H22 ! dihe -179.7891

DIHE C17 C18 C19 C20 ! dihe -0.1744

DIHE H19 C18 C19 C20 ! dihe 179.9342

DIHE C17 C18 C19 H20 ! dihe 179.8817

DIHE H19 C18 C19 H20 ! dihe -0.0098

DIHE C18 C19 C20 C21 ! dihe 0.0833

DIHE H20 C19 C20 C21 ! dihe -179.9715

DIHE C18 C19 C20 F1 ! dihe -179.9848

DIHE H20 C19 C20 F1 ! dihe -0.0396

DIHE C19 C20 C21 C22 ! dihe 0.0473

DIHE F1 C20 C21 C22 ! dihe -179.8847

DIHE C19 C20 C21 H21 ! dihe 179.8181

DIHE F1 C20 C21 H21 ! dihe -0.1139

DIHE C20 C21 C22 C17 ! dihe -0.0891

DIHE H21 C21 C22 C17 ! dihe -179.8548

DIHE C20 C21 C22 H22 ! dihe 179.7081

DIHE H21 C21 C22 H22 ! dihe -0.0575

IMPH C8 C6 C1 C2

IMPH C1 C3 C2 H1

IMPH C2 C4 C3 H2

IMPH C3 C5 C4 H3

IMPH C4 C6 C5 H4

IMPH C1 C5 C6 N1

IMPH C7 C6 N1 H5

IMPH N1 N2 C7 O2

IMPH C7 C8 N2 C9

IMPH C1 N2 C8 O1

IMPH C13 C17 C16 O3

IMPH C16 C18 C17 C22

IMPH C17 C19 C18 H19

IMPH C18 C20 C19 H20

IMPH C19 C21 C20 F1

IMPH C20 C22 C21 H21

IMPH C17 C21 C22 H22

***Parameter file for KET***

* Force Field Parameter File.

*

BOND

lca lca 478.40 1.387

lc lca 349.70 1.487

lca lha 344.30 1.087

lca ln 372.30 1.422

lc ln 478.20 1.345

lhn ln 410.20 1.009

lc lo 648.00 1.214

lc3 ln 330.60 1.460

lc3 lc3 303.10 1.535

lc3 lh1 335.90 1.093

lc3 ln4 293.60 1.499

lc3 lhx 338.70 1.091

lhn ln4 369.00 1.033

lc3 lhc 337.30 1.092

lc lc3 328.30 1.508

lca lf 363.80 1.344

ANGLE

lca lca lca 67.200 119.970

lca lca lha 48.500 120.010

lca lca ln 68.000 119.890

lca lc ln 69.400 112.030

lca lc lo 68.700 123.440

lca lca lc 64.600 120.140

lca ln lc 64.300 123.710

lca ln lhn 47.600 114.590

ln lc ln 75.400 111.700

ln lc lo 75.800 122.030

lc ln lhn 49.200 118.460

lc ln lc 67.400 119.630

lc ln lc3 63.900 121.350

ln lc3 lc3 65.900 112.130

ln lc3 lh1 49.800 109.320

lc3 lc3 ln4 66.000 108.930

lc3 lc3 lhx 46.000 111.740

lc3 lc3 lh1 46.400 110.070

lc3 ln4 lc3 62.800 110.640

lc3 ln4 lhn 46.200 110.110

ln4 lc3 lhx 49.000 107.910

lc3 lc3 lc3 63.200 110.630

lc3 lc3 lhc 46.400 110.050

lc3 lc3 lc 63.800 110.530

lc3 lc lo 68.000 123.110

lc3 lc lca 62.300 119.530

lc lc3 lhc 47.200 109.680

lca lca lf 67.600 118.740

lh1 lc3 lh1 39.200 109.550

lhx lc3 lhx 39.000 110.740

lhc lc3 lhc 39.400 108.350

DIHEDRAL

X lca lca X 3.625 2 180.0

X lc lca X 3.625 2 180.0

X lca ln X 0.450 2 180.0

X lc ln X 2.500 2 180.0

lhn ln lc lo 2.500 2 180.0

lhn ln lc lo 2.000 1 0.0

X lc3 ln X 0.000 2 0.0

lc3 lc3 ln lc 0.500 4 180.0

lc3 lc3 ln lc 0.150 3 180.0

lc3 lc3 ln lc 0.530 1 0.0

X lc3 lc3 X 0.156 3 0.0

X lc3 ln4 X 0.156 3 0.0

lc3 lc3 lc3 lc3 0.180 3 0.0

lc3 lc3 lc3 lc3 0.250 2 180.0

lc3 lc3 lc3 lc3 0.200 1 180.0

lhc lc3 lc3 lc3 0.160 3 0.0

lhc lc3 lc3 lhc 0.150 3 0.0

X lc lc3 X 0.000 2 180.0

lhc lc3 lc lo 0.800 1 0.0

lhc lc3 lc lo 0.080 3 180.0

IMPHI

lc lca lca lca 1.100 0 180.0 ! Using delfault value

X X lca lha 1.100 0 180.0

lca lca lca ln 1.100 0 180.0 ! Using delfault value

X X ln lhn 1.100 0 180.0

X X lc lo 10.500 0 180.0

lc lc ln lc3 1.100 0 180.0 ! Using delfault value

lca lca lca lf 1.100 0 180.0

NONBONDED NBXMOD 5 GROUP SWITCH CDIEL -

CUTNB 14.0 CTOFNB 12.0 CTONNB 10.0 EPS 1.0 E14FAC 0.83333333 WMIN 1.4

! Emin Rmin/2 Emin/2 Rmin (lfor 1-4's)

! (klcal/mol) (A)

lca 0.00 -0.0860 1.9080 0.00 -0.0430 1.9080

ln 0.00 -0.1700 1.8240 0.00 -0.0850 1.8240

lc 0.00 -0.0860 1.9080 0.00 -0.0430 1.9080

lo 0.00 -0.2100 1.6612 0.00 -0.1050 1.6612

lc3 0.00 -0.1094 1.9080 0.00 -0.0547 1.9080

ln4 0.00 -0.1700 1.8240 0.00 -0.0850 1.8240

lf 0.00 -0.0610 1.7500 0.00 -0.0305 1.7500

lha 0.00 -0.0150 1.4590 0.00 -0.0075 1.4590

lhn 0.00 -0.0157 0.6000 0.00 -0.0078 0.6000

lh1 0.00 -0.0157 1.3870 0.00 -0.0078 1.3870

lhx 0.00 -0.0157 1.1000 0.00 -0.0078 1.1000

lhc 0.00 -0.0157 1.4870 0.00 -0.0078 1.4870

**Supplemental References**

1. Shan J, Weinstein H, Mehler EL (2010) Probing the Structural Determinants for the Function of Intracellular Loop 2 in Structurally Cognate G-Protein-Coupled Receptors. Biochemistry 49: 10691-10701.

2. Kortagere S, Roy A, Mehler EL (2006) Ab initio computational modeling of long loops in G-protein coupled receptors. J Comput Aided Mol Des 20: 427-436.

3. Mehler EL, Hassan SA, Kortagere S, Weinstein H (2006) *Ab initio* computational modeling of loops in G-protein-coupled receptors: lessons from the crystal structure of rhodopsin. Proteins 64: 673-690.

4. Mehler EL, Periole X, Hassan SA, Weinstein H (2002) Key issues in the computational simulation of GPCR function: representation of loop domains. J Comput Aided Mol Des 16: 841-853.

5. Okada T, Sugihara M, Bondar AN, Elstner M, Entel P, et al. (2004) The retinal conformation and its environment in rhodopsin in light of a new 2.2 Å crystal structure. J Mol Biol 342: 571-583.

6. Cherezov V, Rosenbaum DM, Hanson MA, Rasmussen SG, Thian FS, et al. (2007) High-resolution crystal structure of an engineered human β_2_-adrenergic G protein-coupled receptor. Science 318: 1258-1265.

7. Ballesteros JA, Weinstein H (1995) Integrated Methods for Modeling G-Protein Coupled Receptors. Methods Neurosci 25: 366-428.

8. Strader CD, Sigal IS, Dixon RA (1989) Structural basis of beta-adrenergic receptor function. FASEB J 3: 1825-1832.

9. Lan H, Liu Y, Bell MI, Gurevich VV, Neve KA (2009) A dopamine D2 receptor mutant capable of G protein-mediated signaling but deficient in arrestin binding. Mol Pharmacol 75: 113-123.

10. Bouvier M, Collins S, O'Dowd BF, Campbell PT, de Blasi A, et al. (1989) Two distinct pathways for cAMP-mediated down-regulation of the beta 2-adrenergic receptor. Phosphorylation of the receptor and regulation of its mRNA level. J Biol Chem 264: 16786-16792.

11. Bouvier M, Guilbault N, Bonin H (1991) Phorbol-ester-induced phosphorylation of the beta 2-adrenergic receptor decreases its coupling to Gs. FEBS Lett 279: 243-248.

12. Shapiro RA, Nathanson NM (1989) Deletion analysis of the mouse m1 muscarinic acetylcholine receptor: effects on phosphoinositide metabolism and down-regulation. Biochemistry 28: 8946-8950.

13. Keefer JR, Kennedy ME, Limbird LE (1994) Unique structural features important for stabilization versus polarization of the alpha 2A-adrenergic receptor on the basolateral membrane of Madin-Darby canine kidney cells. J Biol Chem 269: 16425-16432.

14. Schoneberg T, Liu J, Wess J (1995) Plasma membrane localization and functional rescue of truncated forms of a G protein-coupled receptor. J Biol Chem 270: 18000-18006.

15. Kobilka BK, Kobilka TS, Daniel K, Regan JW, Caron MG, et al. (1988) Chimeric alpha 2-,beta 2-adrenergic receptors: delineation of domains involved in effector coupling and ligand binding specificity. Science 240: 1310-1316.

16. Saunders C, Limbird LE (2000) Microtubule-dependent regulation of alpha(2B) adrenergic receptors in polarized MDCKII cells requires the third intracellular loop but not G protein coupling. Mol Pharmacol 57: 44-52.

17. Jaakola VP, Vainio M, Sen S, Rehn M, Heimo H, et al. (2005) Intracellularly truncated human alpha2B-adrenoceptors: stable and functional GPCRs for structural studies. J Recept Signal Transduct Res 25: 99-124.

18. Warne T, Serrano-Vega MJ, Baker JG, Moukhametzianov R, Edwards PC, et al. (2008) Structure of a β_1_-adrenergic G-protein-coupled receptor. Nature 454: 486-491.

19. Grossfield A, Feller SE, Pitman MC (2006) A role for direct interactions in the modulation of rhodopsin by omega-3 polyunsaturated lipids. Proc Natl Acad Sci U S A 103: 4888-4893.

20. Khelashvili G, Grossfield A, Feller SE, Pitman MC, Weinstein H (2009) Structural and dynamic effects of cholesterol at preferred sites of interaction with rhodopsin identified from microsecond length molecular dynamics simulations. Proteins 76: 403-417.
